# The Parkinson’s Disease-Associated Protein Kinase LRRK2 Modulates Notch Signaling through the Endosomal Pathway

**DOI:** 10.1371/journal.pgen.1005503

**Published:** 2015-09-10

**Authors:** Yuzuru Imai, Yoshito Kobayashi, Tsuyoshi Inoshita, Hongrui Meng, Taku Arano, Kengo Uemura, Takeshi Asano, Kenji Yoshimi, Chang-Liang Zhang, Gen Matsumoto, Toshiyuki Ohtsuka, Ryoichiro Kageyama, Hiroshi Kiyonari, Go Shioi, Nobuyuki Nukina, Nobutaka Hattori, Ryosuke Takahashi

**Affiliations:** 1 Department of Research for Parkinson's Disease, Juntendo University Graduate School of Medicine, Tokyo, Japan; 2 Department of Neurology, Graduate School of Medicine, Kyoto University, Kyoto, Japan; 3 CREST (Core Research for Evolutionary Science and Technology), Japan Science and Technology Agency, Saitama, Japan; 4 Research Institute for Diseases of Old Age, Juntendo University Graduate School of Medicine, Tokyo, Japan; 5 Department of Neurophysiology, Juntendo University Graduate School of Medicine, Tokyo, Japan; 6 Department of Neuroscience for Neurodegenerative Disorders, Juntendo University Graduate School of Medicine, Tokyo, Japan; 7 Department of Cell Biology, Institute for Virus Research, Kyoto University, Kyoto, Japan; 8 Laboratory for Animal Resources and Genetic Engineering, RIKEN Center for Developmental Biology, Kobe, Japan; 9 Department of Neurology, Juntendo University Graduate School of Medicine, Tokyo, Japan; National University of Singapore, SINGAPORE

## Abstract

Leucine-rich repeat kinase 2 (LRRK2) is a key molecule in the pathogenesis of familial and idiopathic Parkinson’s disease (PD). We have identified two novel LRRK2-associated proteins, a HECT-type ubiquitin ligase, HERC2, and an adaptor-like protein with six repeated Neuralized domains, NEURL4. LRRK2 binds to NEURL4 and HERC2 via the LRRK2 Ras of complex proteins (ROC) domain and NEURL4, respectively. HERC2 and NEURL4 link LRRK2 to the cellular vesicle transport pathway and Notch signaling, through which the LRRK2 complex promotes the recycling of the Notch ligand Delta-like 1 (Dll1)/Delta (Dl) through the modulation of endosomal trafficking. This process negatively regulates Notch signaling through *cis*-inhibition by stabilizing Dll1/Dl, which accelerates neural stem cell differentiation and modulates the function and survival of differentiated dopaminergic neurons. These effects are strengthened by the R1441G ROC domain-mutant of LRRK2. These findings suggest that the alteration of Notch signaling in mature neurons is a component of PD etiology linked to *LRRK2*.

## Author Summary

LRRK2 is linked to autosomal dominant late-onset Parkinson’s disease, suggesting that LRRK2 gain-of-function mutations lead to age-dependent degeneration of the midbrain dopaminergic neurons. In this study, we describe two novel LRRK2-associated proteins HERC2 and NEURL4, which are a ubiquitin ligase and an adaptor-like protein, respectively. HERC2 and NEURL4 direct LRRK2 to Notch signaling pathway, in which the LRRK2-NEURL4-HERC2 complex promotes the recycling of the Notch ligand Delta-like 1 (Dll1)/Delta (Dl) through the modulation of endosomal trafficking. As a result, the amounts of Dll1/D1 on the plasma membrane are increased, which affects negatively Notch signaling through *cis*-inhibition. The effect is enhanced by a Parkinson’s-disease associated mutation of LRRK2. Inhibition of Notch signaling in adult dopaminergic neurons impairs its functions and survival. These findings indicate a possible link between Notch pathway and Parkinson’s disease.

## Introduction

Parkinson’s disease (PD) is thought to result from the combined effects of environmental and genetic factors. *LRRK2*, which encodes a ROCO protein with a Ras of complex proteins (ROC) domain, a conserved C-terminal of ROC (COR) domain, a kinase domain, leucine-rich repeats (LRRs), and WD40 repeats, has been identified as a causative gene for autosomal-dominant familial PD [1]. Although various amino acid substitutions have been identified throughout the multiple domains of LRRK2, including R1441G (RG) in the ROC domain and G2019S (GS) and I2020T (IT) in the kinase domain, the pathogenic role of LRRK2 mutations and the biochemical pathways involved remain largely unknown. *LRRK2*-associated familial PD is almost indistinguishable from the common sporadic form of PD in clinical and pathological aspects. *LRRK2* mutations identified in familial PD patients have also been isolated in 1–5% of PD patients without apparent familial PD histories. Moreover, two recent genome-wide-association studies (GWAS) detected *LRRK2* and *SNCA*/*a-synuclein* as two strong risk loci for sporadic PD [2]. The single-nucleotide polymorphisms (SNPs) in *LRRK2* were situated in the non-coding region, upstream of the *LRRK2* coding sequence, which suggests that even certain changes in the expression level of wild-type (WT) LRRK2, presumably incremental, could increase the risk of PD. Consistent with this possibility, experimental data from α-synuclein and LRRK2 double-transgenic mice suggest that the difference in LRRK2 protein expression levels is more important than LRRK2 kinase activity [3].

LRRK2 is highly expressed in the regions of active cell differentiation, migration and cell death during the development of the mouse embryo [4]. Loss of the *LRRK2* gene in mice has been demonstrated to impair the autophagy-lysosome pathway, which leads to marked accumulations of α-Synuclein and ubiquitinated proteins [5] or enlarged and increased numbers of secondary lysosomes in the kidney with age [6]. A single *LRRK* gene (referred to hereafter as *dLRRK*) was identified in the *Drosophila* genome. Critical residues disrupted in familial PD are conserved between LRRK2 and dLRRK, although dLRRK structurally resembles LRRK1 [7, 8]. dLRRK is localized in the endosomes and has also been reported to regulate the function of Rab7 in the endosomal-lysosomal pathway [9, 10].

Notch is a large transmembrane receptor that is activated by ligand binding. The signaling pathway of Notch is an evolutionarily well-conserved signaling system and is known for its important roles in development, such as determining the timing and direction of cellular differentiation and the development and maintenance of borders in developing tissues. Numerous studies have revealed the importance of Notch in the nervous system, including in the maintenance of immature neurons, the control of neurite outgrowth of differentiated neurons, and the regulation of synaptic plasticity and olfactory functions in the adult brain [11–14].

Binding between Notch and Notch ligands on contacting cells results in the proteolytic cleavage of Notch at the transmembrane region and the subsequent release of the Notch intracellular domain (NICD). NICD then translocates into the nucleus and functions as a transcriptional activator for its target genes, such as *Hes1* and *Hes5*, along with additional transcriptional factors. Notch signaling is regulated in multiple ways, such as glycosyl modification of Notch, *cis-trans* regulation by ligands, and endocytic trafficking and degradation of Notch signal components [15]. On a given cell, Notch can be *trans*-activated by Notch ligands presented by neighboring cells, whereas Notch ligands in the same cell have a cell-autonomous dominant-negative effect on the Notch receptor. This regulatory mechanism is called the ‘*cis*-inhibition’ of Notch signaling [16] and is thought to ensure polarized signaling in cell-cell communications.

Here, we report two novel LRRK2-binding proteins, NEURL4 and HERC2, both of which genetically and physically interact with the Notch ligand Dll1/Dl. Our data reveal that, in cooperation with NEURL4 and HERC2, LRRK2 modulates the Notch signaling through the regulation of the trafficking of Dll1/Dl, by which the function and survival of adult dopaminergic neurons are altered. Therefore, abnormal Notch signaling could be a component of the mechanism of neurodegeneration in PD.

## Results

### HERC2 and NEURL4 as novel LRRK2-binding proteins

**Fig 1:**
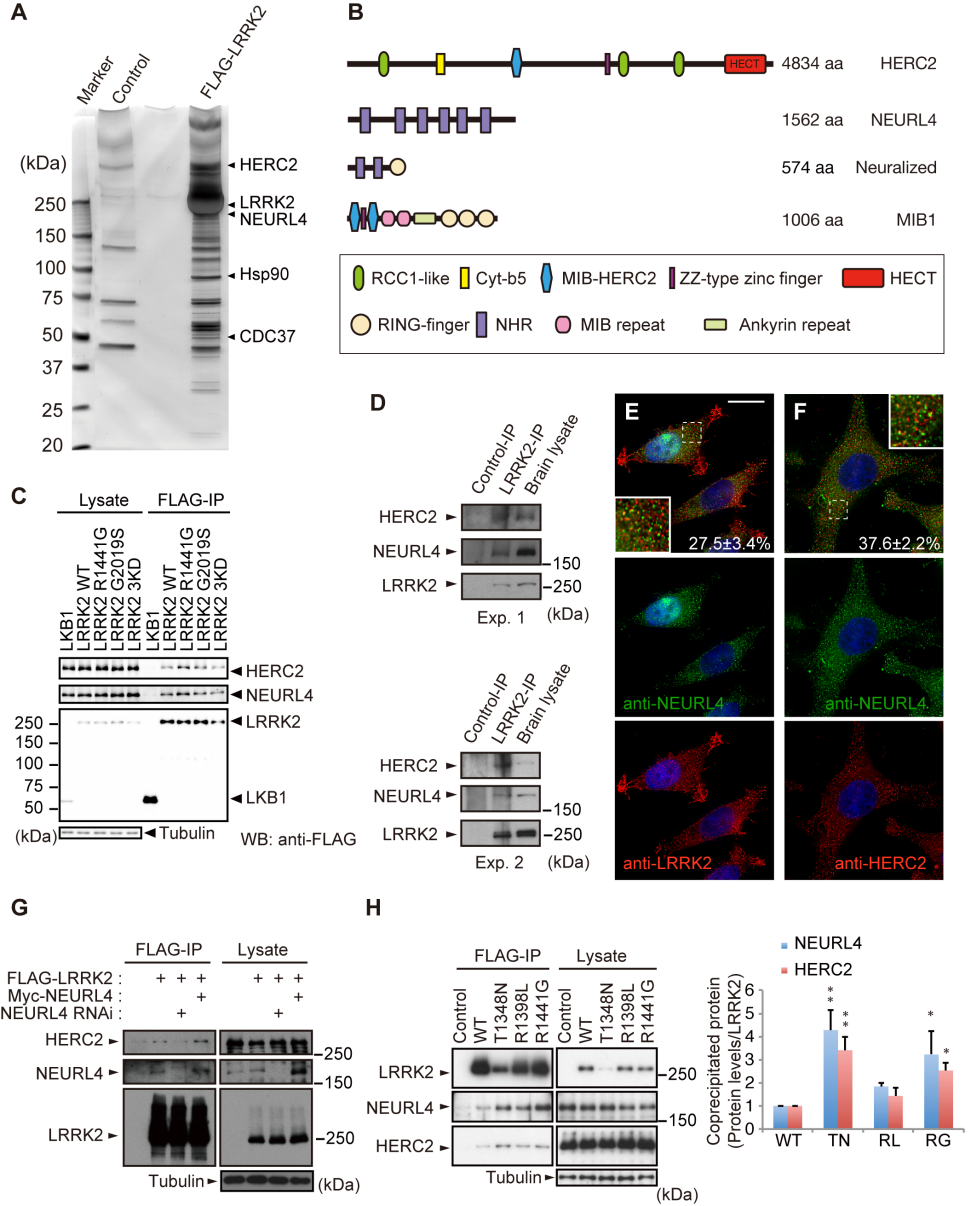
Identification of HERC2 and NEURL4 as LRRK2-binding proteins. (A) Silver-stained gel image for LRRK2-binding proteins. LRRK2 and representative co-purified proteins are indicated. (B) Domain structures of HERC2, NEURL4 and their related proteins Neuralized and Mindbomb homolog 1 (MIB1). RCC1-like, Regulator of chromosome condensation 1-like domain; Cyt-b5, cytochrome b5-like motif; MIB-HERC2, MIB/HERC2 domain; NHR, Neuralized homology repeat domain. (C) HERC2 and NEURL4 specifically bind to LRRK2 in HEK293T cells. Lysates expressing FLAG-tagged LRRK2 or FLAG-tagged LKB1 were subjected to immunoprecipitation with anti-FLAG antibody (FLAG-IP) and were detected by Western blotting with anti-FLAG, anti-HERC2 and anti-NEURL4 antibodies. (D) Endogenous associations between LRRK2, NEURL4 and HERC2. Mouse brain lysates were subjected to immunoprecipitation with anti-LRRK2 (LRRK2-IP) or anti-dFoxO (Control-IP) antibodies as a control and analyzed by Western blotting with the indicated antibodies. The results of two independent experiments are shown. (E) LRRK2, NEURL4 and HERC2 are partially colocalized as vesicular signals in the cytosol. HeLa cells transfected with LRRK2 were visualized with anti-NEURL4 (green) and anti-LRRK2 (red) antibodies, and the nuclei were counterstained with DAPI (blue). Colocalization of the proteins appears as yellow. Insets show higher-magnification images of the boxed regions and the values ± SE in (E, F) indicates colocalized green signals with red (n = 4). Scale bar, 10 μm. (F) HeLa cells transfected with HERC2 were visualized with anti-NEURL4 (green) and anti-HERC2 (red) antibodies as in (E). (G) LRRK2 binds to HERC2 via NEURL4. HEK293T cell lysates transfected with FLAG-LRRK2, Myc-NEURL4, an siRNA duplex against NEURL4 and/or an empty plasmid were subjected to immunoprecipitation with an anti-FLAG antibody and detected by Western blotting with antibodies against the indicated proteins. (H) LRRK2 GTPase mutations (TN, T1348N; RL, R1398L; RG, R1441G) modulate the affinity to NEURL4 and HERC2. Note that LRRK2^TN^ consistently exhibited lower expression levels, suggesting instability. The graph indicates the relative levels of endogenous proteins co-precipitated with FLAG-LRRK2. The data are shown as the mean ± SE from five repeated experiments (**, *p* < 0.01; *, *p* < 0.05 by one-way ANOVA).

To investigate the functions of LRRK2, we searched for novel LRRK2-binding proteins. N-terminally FLAG-tagged LRRK2 was expressed in HEK293 cells, and the LRRK2-associated protein complexes were affinity-purified from the cell lysate using anti-FLAG-conjugated beads. Of the binding proteins detected by liquid chromatography/tandem mass spectrometry (LC-MS/MS), we chose two novel proteins, HERC2 and NEURL4, for further investigation (Fig 1A).

HERC2 is a large protein with a predicted molecular mass of more than 500 kDa. HERC2 has three RCC1-like domains (RLDs), a mind-bomb (MIB)/HERC2 domain, and a C-terminal HECT domain (Fig 1B). NEURL4 includes six Neuralized homology repeat (NHR) domains, which have been reported to associate with HERC2 and to regulate centrosome duplication (Fig 1B) [17–19].

Co-immunoprecipitation using the FLAG-tagged proteins confirmed that endogenous HERC2 and NEURL4 bind to FLAG-LRRK2, including pathogenic and kinase-dead (KD) mutants, but not to the unrelated LKB1-FLAG in HEK293 cells (Fig 1C). Physiological binding of these proteins was also detected by the co-immunoprecipitation of endogenous proteins from mouse brain lysates (Fig 1D), and these proteins partially colocalized with each other in cultured cells (Figs 1E, 1F and S1A–S1E). NEURL4 knockdown abolished the binding between HERC2 and LRRK2, and Myc-NEURL4 enhanced Flag-LRRK2 co-immunoprecipitation with HERC2 (Fig 1G), suggesting that LRRK2 binds to HERC2 through NEURL4. The binding of LRRK2 to NEURL4 was exclusively dependent on the ROC domain and partly on the WD40 domain (S1F, S1G and S1I Fig). The LRRK2 mutant LRRK2^RG^, a ROC domain pathogenic mutant, and LRRK2^TN^, an artificial mutant with impaired GTP-binding activity, stabilized the binding to HERC2 and NEURL4 (Fig 1H), whereas LRRK2^GS^, a kinase domain pathogenic mutant, and LRRK2^RL^, an artificial mutant with increased GTP hydrolysis activity, did not affect that binding (Fig 1C and 1H) [20, 21]. NEURL4 bound to LRRK2 through a region containing the third and fourth NHR domains of NEURL4 (S1H and S1J Fig). HERC2 bound to NEURL4 through a region containing the fifth and sixth NHR domains of NEURL4 (S1H and S1J Fig).

### LRRK2 binds to Dll1 through NEURL4 and HERC2

**Fig 2:**
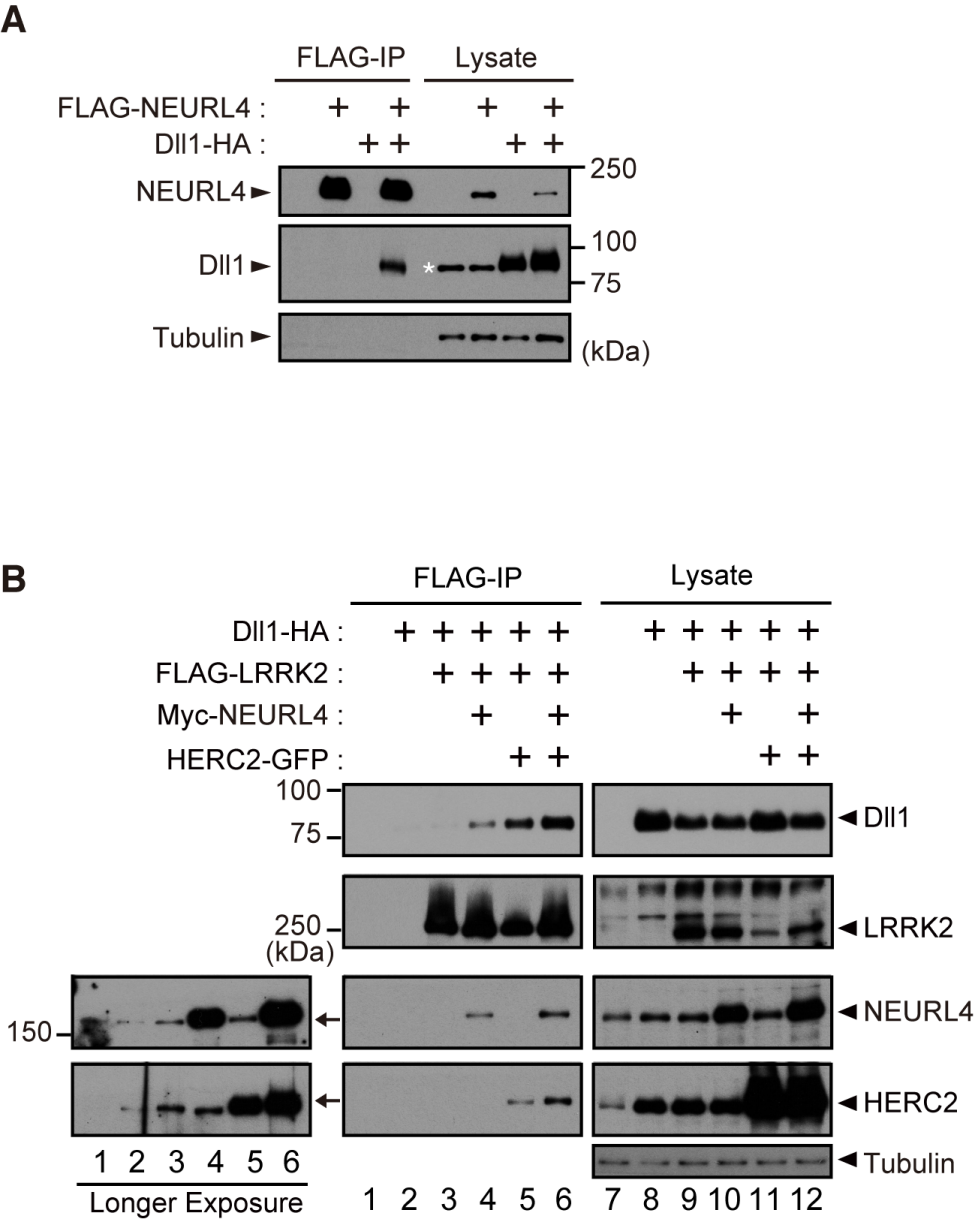
LRRK2, NEURL4 and HERC2 bind to the Notch ligand Dll1. (A) NEURL4 binds to Dll1. HEK293T cell lysate transfected with the indicated plasmids was subjected to immunoprecipitation with an anti-FLAG antibody and analyzed by Western blotting with anti-FLAG and anti-HA antibodies. The asterisk indicates non-specific bands that appeared with anti-HA (clone 12CA5). (B) LRRK2 associates with Dll1 via NEURL4 and HERC2. A co-immunoprecipitation assay was performed as in (A). Western blotting was performed with anti-Dll1, anti-LRRK2, anti-NEURL4 and anti-HERC2 antibodies. Longer exposures of X-ray films revealed endogenous proteins that co-immunoprecipitate with FLAG-LRRK2 (Left).

Domain analysis of HERC2 and NEURL4 (Fig 1B) suggested that both proteins might be involved in the Notch signaling pathway. HERC2 has an MIB/HERC2 domain, and NEURL4 has four NHR domains. Neur, which is a ubiquitin-ligase for Dl, has been shown in *Drosophila* to recognize Dl through the NHR1 domain [22], and the MIB/HERC2 domain is also included in the Dl-binding region of MIB, another ubiquitin-ligase for Dl [23]. As expected, NEURL4 binds to the mammalian Dl homologue Dll1 (Fig 2A). In addition, NEURL4 also binds to Neur (S2A Fig). However, NEURL4 did not compete with Neur in the binding to Dll1; instead, NEURL4 enhanced the binding of Neur to Dll1 (S2B Fig). LRRK2 exhibited little binding with Dll1, whereas both NEURL4 and HERC2 enhanced the binding of LRRK2 to Dll1 (Fig 2B). Moreover, an immunoprecipitation assay indicated that LRRK2, NEURL4 and HERC2 specifically bound to several types of Rabs (S2C–S2E Fig). These results suggested that NEURL4 and HERC2 assist LRRK2 in dynamically regulating Dll1 in the endosomes.

### LRRK2, NEURL4 and HERC2 genetically interact with Dl in the fly

**Fig 3:**
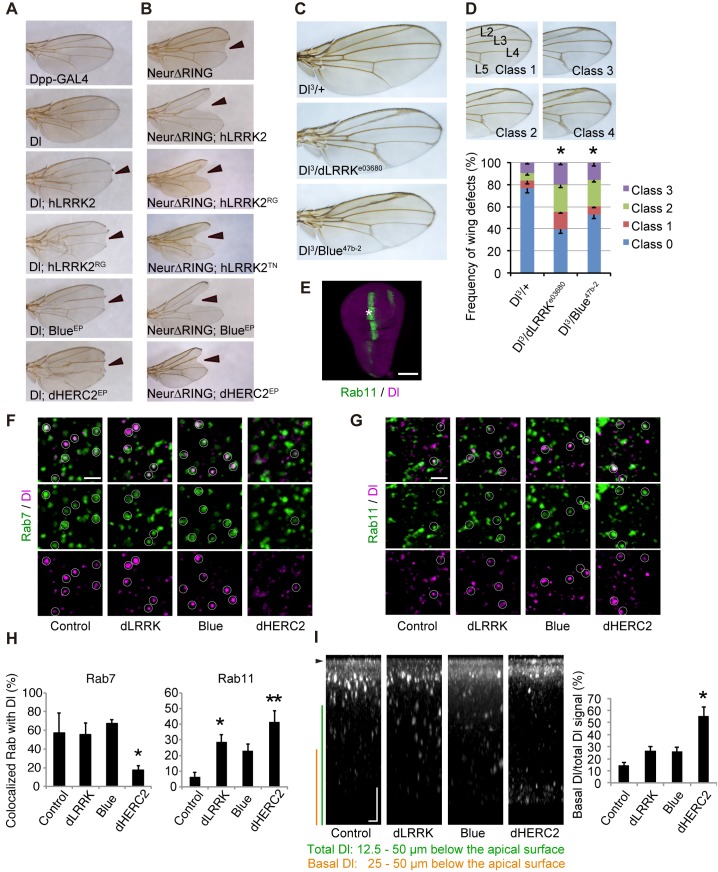
*LRRK2*, *HERC2* and *NEURL4* genetically interact with Dl signaling components in *Drosophila*. (A) The *Dpp-GAL4* driver by itself produced normal wing morphology. A low level *Dl* expression causes a mild defect in the vein formation. *hLRRK2*, *hLRRK2* ^*RG*^, *Blue* or *dHERC2* co-expression together with *Dl* produces notched wing margins (arrowheads). EP lines were used for *Blue* and *dHERC2* overexpression. (B) *hLRRK2*, *Blue* or *dHERC2* exacerbates the phenotype of the dominant-negative *Neur*. Overexpression of *Neur* lacking the RING-finger domain (Dl-∆RING) influences the wing margin formation (arrowheads), and this effect is enhanced by *hLRRK2*, *Blue* or *dHERC2* co-expression. (C, D) The *Dl* phenotype is enhanced by the reduced activity of dLRRK or Blue. *Dl*, *dLRRK* and *Blue* loss-of-function alleles were combined, and the wing phenotypes of (C) were quantified using a scoring system (D). Classification used to quantify frequencies of *Dl* phenotypes: Class 0, normal; Class 1, mild thickening of the longitudinal vein L2; Class 2, moderate thickening of the L2 and L5 veins and the distal tip of L3 and L4 veins; and Class 3, severe thickening of the L2-L5 veins. The data are shown as the mean ± SE from three experiments. * *p* < 0.05 *vs*. *Dl* ^*3*^ */+* by Steel-Dwass test. n = 108–200. *Dpp-GAL4* drove all transgenes at 18°C (A) or 25°C (B-D). (E-H) Both dLRRK and dHERC2 promote Dl localization at the recycling endosomes. Colocalized Dl signals with Rab7 (circles in F) or Rab11 (circles in G) in the developing L3 vein region of the wing disc (asterisk in E) were imaged and graphed (H). Scale bars, 100 μm in E and 2 μm in F, G. (I) The LRRK2 complex does not inhibit Dl endocytosis. Images show the distribution of internalized anti-Dl in the confocal z-sections, and dHERC2 promotes Dl transcytosis to the basal direction. The graph indicates the basal distribution of the Dl signal. Arrowhead, apical surface. Scale bars on X/Z-axes, 2/10 μm. Graphs in (H, I) are shown as the mean ± SE. **, *p* < 0.01; *, *p* < 0.05 by Dunnett’s test. n = 3–6.

We next examined the influence of LRRK2, NEURL4 and HERC2 on Notch signaling in *Drosophila*. During wing development in *Drosophila* larvae, the future dorso-ventral border in the wing imaginal disc is maintained by the activity of Notch signaling along the border. Attenuation of Notch signaling in the border results in the diminution of the border, and the phenotype of the “*notched*” wing will be present in the adult fly. We utilized the *Dpp-Gal4>UAS* system, in which the *Dpp-GAL4* driver drives transgenes with upstream activating sequences (UASs) in a temperature-dependent manner. The *Dpp* promoter is active along the long axis of the wing disc, orthogonally to the dorso-ventral border. *Dpp-GAL4* expression had no effects on wing development (Fig 3A, Dpp-Gal4). *Drosophila* has single copies of homologous genes for *LRRK2*, *NEURL4* and *HERC2*, which are *dLRRK*, *Bluestreak (Blue)* and *dHERC2*, respectively. Ectopic expression or knockdown of *dLRRK*, *Blue* or *dHERC2* alone had no effects on wing formation. Ectopic expression of Dl along the long axis of the wing disc using *Dpp-GAL4* resulted in a shrunken and completely deformed wing at 25°C. Reducing the expression of Dl by raising fly crosses at 18°C masked the wing phenotype (Fig 3A, Dl). However, the co-expression of human LRRK2 (Fig 3A, Dl; hLRRK2), Blue (Fig 3A, Dl; Blue^EP^, and S3A and S3B Fig) or dHERC2 (Figs 3A, Dl; dHERC2^EP^, and S3C) with Dl at 18°C caused “notching” of the wing margin. Expression of the pathogenic mutant LRRK2^RG^ produced a similar but stronger phenotype (Fig 3A, Dl; hLRRK2^RG^) compared with the LRRK2 wild-type (WT) crosses (Fig 3A, Dl; hLRRK2). These results suggested that ectopic expression of LRRK2, NEURL4 and HERC2 compromises Notch signaling through Dl modulation.

Neur lacking its RING-finger domain (Neur∆RING) acts as a dominant-negative, and its expression results in Dl accumulation on the cell surface and the appearance of the wing phenotype by *cis*-inhibition (Fig 3B). *LRRK2*, *Blue* or *dHERC2* co-expression further enhanced the notching phenotype (Fig 3B), and LRRK2^RG^, LRRK2^TN^, LRRK2^GS^, and LRRK2^KD^ caused a size reduction in Neur∆RING (Figs 3B and S3D). However, the notching phenotype by LRRK2^KD^ was milder (S3D Fig). The loss of dLRRK partly suppressed the notching phenotype by Neur∆RING (S3D Fig). Full-length Neur expression had little effect on wing development, whereas co-expression of dHERC2 led to wing size reduction (S3E Fig). Based on the genetic results of dHERC2 in Figs 3B and S3E, Neur and HERC2 appear to act differently rather than in synergy. We also performed a genetic test using loss-of-function alleles of *Dl*, *dLRRK* and *Blue* to substantiate our findings (Fig 3C and 3D). Approximately twenty-five per cent of *Dl* ^*3*^ heterozygous flies exhibited the vein thickening phenotype to varying degrees, which was potentiated by a combination of *dLRRK* (approximately 60%) and *Blue* (approximately 47%) mutant alleles (Fig 3D). Our genetic tests revealed that the LRRK2 complex does not modulate phenotypes caused by the overexpression of Serrate, another ligand for Notch in *Drosophila* (S3F Fig), or by a mutation of Notch (S3G Fig), implying that the effects of the LRRK2 complex are specific to Dl/Dll1. These genetic results suggest that *LRRK2*, *Blue* and *dHERC2* potentiate ‘*cis*-inhibition’, presumably by promoting the accumulation of endogenous Dl on the cell surface of Notch expressing (signal-receiving) cells as well as of signal-sending cells (Fig 3A and 3B) whereas the loss of *dLRRK* or *Blue* results in the reduction of Dl on the cell surface of signal-sending cells, leading to diminished Notch activation (Fig 3C).

Dl is subjected to endosomal trafficking after endocytosis mediated by ubiquitin ligases, such as Neur and MIB [24]. The small GTPases Rab5, Rab7 and Rab11 regulate early, late and recycling endosomes, respectively, and are known to influence the status of Notch signaling [25, 26]. Because LRRK2/dLRRK, NEURL4 and HERC2 binds to Rab proteins, the endosomal trafficking of Dl is likely regulated by the LRRK2 complex. To monitor the endosomal trafficking of Dl, an anti-Dl antibody uptake assay was performed in living wing imaginal discs expressing Rab7-GFP or Rab11-GFP (Fig 3E–3G) [27]. dHERC2 overexpression suppressed the transport of Dl to Rab7-positive late endosomes, whereas dLRRK and dHERC2 promoted the colocalization of Dl with Rab11-positive recycling endosomes. Blue overexpression also tended to increase the colocalization of Dl with Rab11. The same assay revealed that dLRRK and Blue did not inhibit the endocytosis of Dl but rather tended to promote it (Fig 3I). Interestingly, dHERC2 stimulated the transport of Dl in the basal direction (Fig 3I). In contrast, the LRRK2 complex did not change the distribution of Rab7-GFP and Rab11-GFP. These observations strongly suggest that the LRRK2 complex regulates the endosomal recycling of Dl to maintain the levels of Dl at the cell surface but does not inhibit Dl endocytosis.

### LRRK2, NEURL4 and HERC2 suppress Notch signaling

**Fig 4:**
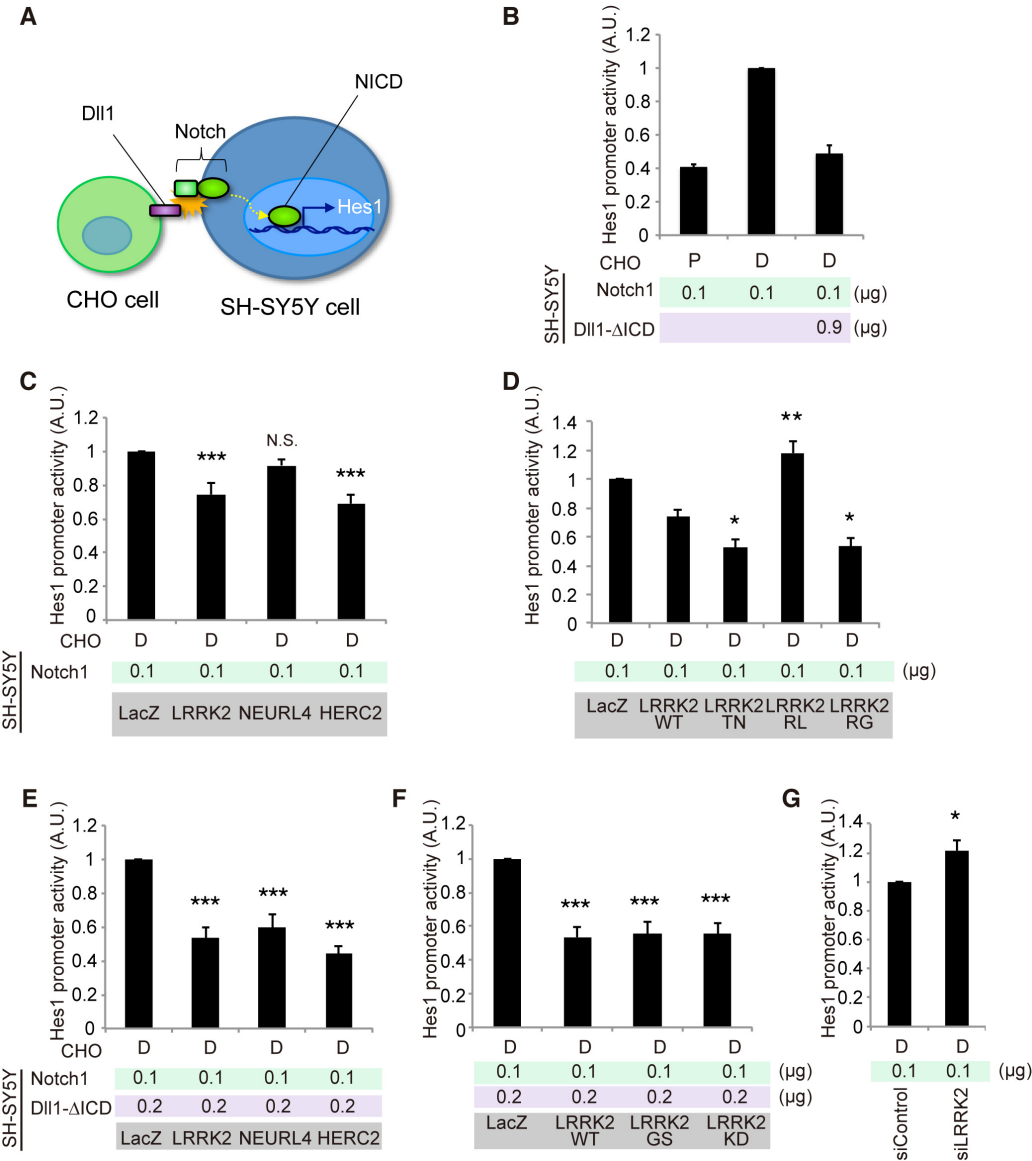
LRRK2, NEURL4 and HERC2 modulate Notch signal intensity in cultured cells. (A) Schematic of non-cell-autonomous Notch signaling and ‘*cis*-inhibition’ reconstituted in cultured cells. (B) Notch signal intensity assessed by the Hes1 promoter activity. SH-SY5Y cells were transfected with Hes1 reporter and Notch1 plasmids and control LacZ or Dll1-∆ICD plasmids. CHO cells stably expressing Dll1 (D) and parental CHO (P) cells were co-cultured as signal-sending and mock cells, respectively. (C) LRRK2 and HERC2 suppress Notch signal intensity. (D) LRRK2 ROC mutations affect the suppressive potency of Notch signaling. (E, F) ‘Weak *cis*-inhibition’ condition accentuates the apparent suppressive effects of LRRK2, NEURL4 and HERC2 on Notch signal intensity. (G) LRRK2 knockdown activates the Notch signal. (C, E, F) ***, *p* < 0.001; **, *p* < 0.01; N.S., not significant *vs*. LacZ and (D) **, *p* < 0.01; *, *p* < 0.05 *vs*. LRRK2 WT by one-way ANOVA. (G) *, *p* < 0.05 by Student’s *t*-test. The data represent the mean ± SE at least from three experiments performed in triplicate. KD, kinase-dead.

We next examined the effects of LRRK2, NEURL4 or HERC2 on non-cell-autonomous Notch signaling using a luciferase-based reporter assay, in which the Hes1 promoter activity was monitored based on Notch signal intensity. We used SH-SY5Y cells as Notch signal-receiving cells and CHO cells stably expressing rat Dll1 or parental CHO cells co-cultured as Notch signal-sending cells (Fig 4A). SH-SY5Y cells express a certain level of endogenous Notch [28]. However, the Hes1 promoter activity was weak under a co-culture condition with CHO cells expressing Dll1, which suggests that the influence of endogenous Notch was minimal compared with the intensity generated by the addition of exogenous Notch1 (S4A Fig). In this setting, the co-expression of Notch1 and Dll1 lacking an intracellular domain (Dll1-∆ICD) effectively exerted a *cis*-inhibition effect, as reported in *Drosophila* (Fig 4B) [29]; thus, the Hes1 activity was suppressed to a level equivalent to that produced by cells co-cultured with parental CHO cells (Fig 4B).

The co-expression of LRRK2 or HERC2, instead of Dll1-∆ICD, moderately mimicked the *cis*-inhibition, whereas NEURL4 co-expression did not (Fig 4C). The *cis*-inhibitory activity of LRRK2 was enhanced by TN and RG mutations but not by the RL mutation (Fig 4D); the suppressive effect of LRRK2 was independent of its kinase activity although the effect of LRRK2^KD^ was slightly milder (S4B Fig). *Cis*-inhibition by a minimal amount of Dll1-∆ICD (0.2 μg; 1/6 of total DNA transfected) was enhanced by combination with LRRK2, NEURL4, or HERC2 (Fig 4E). The extent of inhibition remained similar among the WT, GS and KD forms (Fig 4F), whereas the knockdown of endogenous LRRK2 increased it, suggesting that LRRK2 negatively regulates Notch activity through *cis*-inhibition (Fig 4G).

### The LRRK2 complex regulates the endosomal trafficking of Dll1

**Fig 5:**
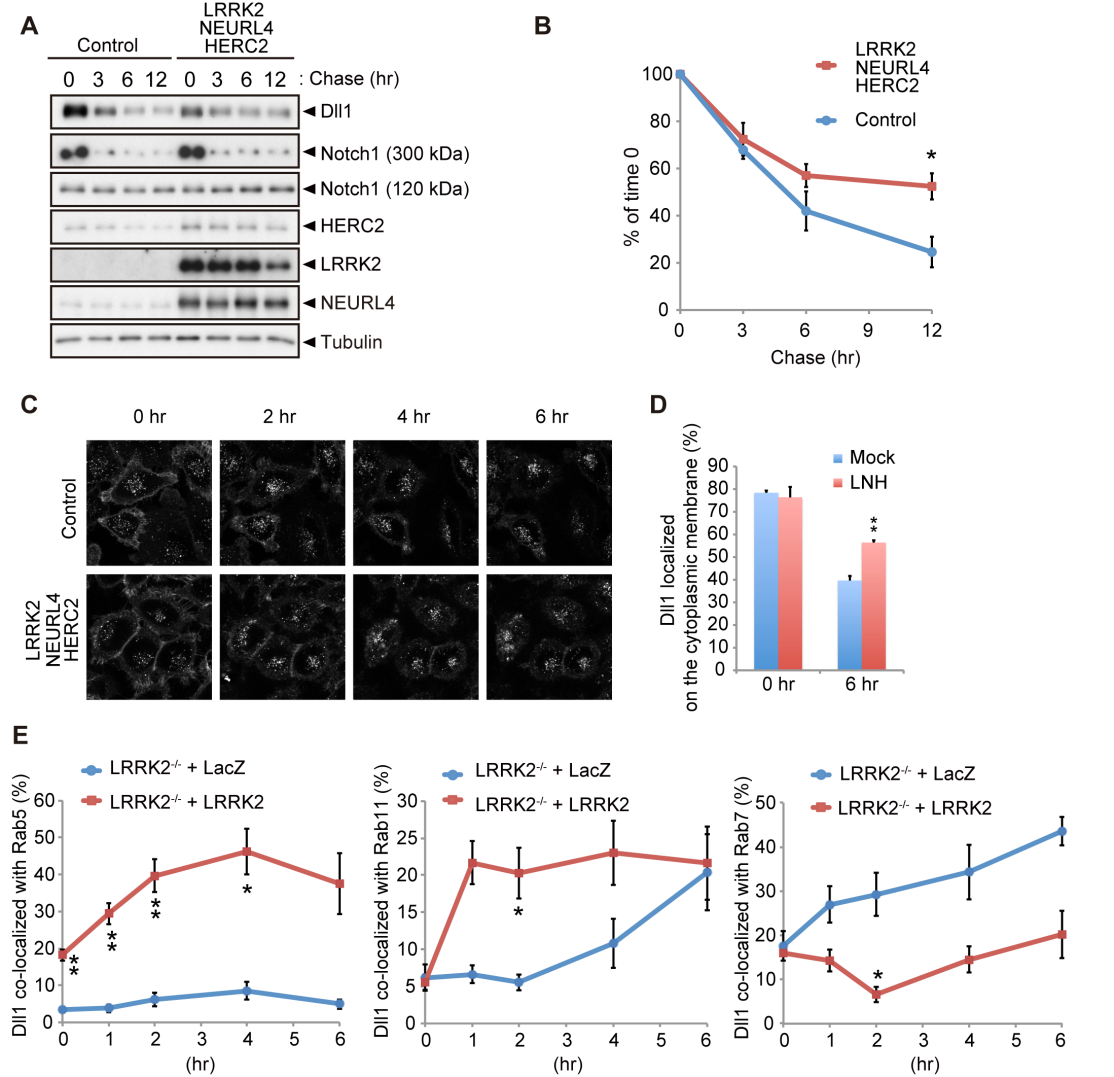
LRRK2 complex modulates Dll1 turnover in the endosomal pathway. (A) Co-expression of LRRK2, NEURL4 and HERC2 stabilizes Dll1. HeLa cells stably expressing Dll1-HA were transfected with a mixture of plasmids for LRRK2/NEURL4/HERC2 or with LacZ as a control. At 24 h after transfection, the cells were then treated with cycloheximide (CHX, 100 μg ml^-1^) for the indicated times, and Western blotting assays were performed. (B) The level of Dll1 remaining at different time points was plotted as the percentage of the initial Dll1 level (0 h of CHX treatment). The data are shown as the mean ± SE from four repeated experiments (*, *p* < 0.05 by Student’s *t*-test). (C) Dynamics of cell surface Dll1. HeLa cells stably expressing Dll1-SNAP were transfected as in (A). Cell surface Dll1 was labeled with SNAP-Surface Alexa Fluor 647 for 20 min at 37°C. Representative grayscale images of Dll1 labeled with Alexa Fluor 647 0–6 h after washout of the tracking dye (t = 0) are shown. (D) LRRK2 complex increases the amount of cell-surface Dll1. The data are presented as the mean ± SE for six independent experiments, with 13–26 cells counted per sample. **, *p* < 0.01 *vs*. Mock at 6 h determined by Student’s *t*-test. (E) LRRK2 stimulates the recycling of Dll1 via the endosomes. *LRRK2*-deficient mouse embryonic fibroblasts stably expressing Dll1-SNAP along with EGFP-Rab5, EGFP-Rab7 or EGFP-Rab11 were transfected with LacZ or LRRK2 and were labeled with SNAP-Surface Alexa Fluor 647 as in (C). Graph showing that Dll1 colocalized with the indicated Rab proteins (mean ± SE for 3–7 independent experiments, with 5–7 cells counted per sample). ** *p* < 0.01, * *p* < 0.05 by Student’s *t*-test.

The *cis*-inhibition by the LRRK2 complex observed in *Drosophila* and a reporter assay using mammalian co-cultured cells was likely caused by alterations in Dll1/Dl turnover or trafficking. The expression of each component or a combination of LRRK2 and NEURL4 did not change the turnover of newly synthesized Dll1 (S5A and S5B Fig), whereas the co-expression of LRRK2 with NEURL4 and HERC2 significantly slowed Dll1 turnover but not that of Notch1 (Figs 5A, 5B and S5C). Next, we explored the specific cellular compartment in which Dll1 was accumulated using a combination of live-cell imaging and SNAP-tag technology. HeLa cells stably expressing Dll1 with a SNAP-tag in the extracellular domain (SNAP-Dll1/HeLa cells) were covalently labeled with a cell-impermeable fluorescent substrate; thus, the dynamics of Dll1 on the cell surface at a specific point in time could be visualized. Internalized Dll1 was incorporated into cellular vesicles, and the cell-surface Dll1 decreased over time (Fig 5C). In this context, Dll1 was expressed more on the cell surface in the LRRK2 complex-expressing cells at 6 h post-labeling (Fig 5D). In contrast, the inactivation of the LRRK2 complex accelerated the disappearance of Dll1 on the plasma membrane (S5D–S5F Fig). To determine the physiological functions of LRRK2 in the endosomal pathway in detail, we used *LRRK2*-deficient cells expressing exogenous LacZ or LRRK2 for Dll1 trafficking. The compensatory expression of LRRK2 increased the colocalization of Dll1 with the Rab5- and Rab11-positive endosomes, whereas it decreased the colocalization of Dll1 with Rab7-positive endosomes (Figs 5E and S6). These findings again suggested that the LRRK2 complex stimulates Dll1 recycling through the endosomal pathway to maintain the cell surface expression of Dll1, which likely explains the enhancement of *cis*-inhibition by the LRRK2 complex.

### The LRRK2 complex accelerates neuronal differentiation in the embryo

**Fig 6:**
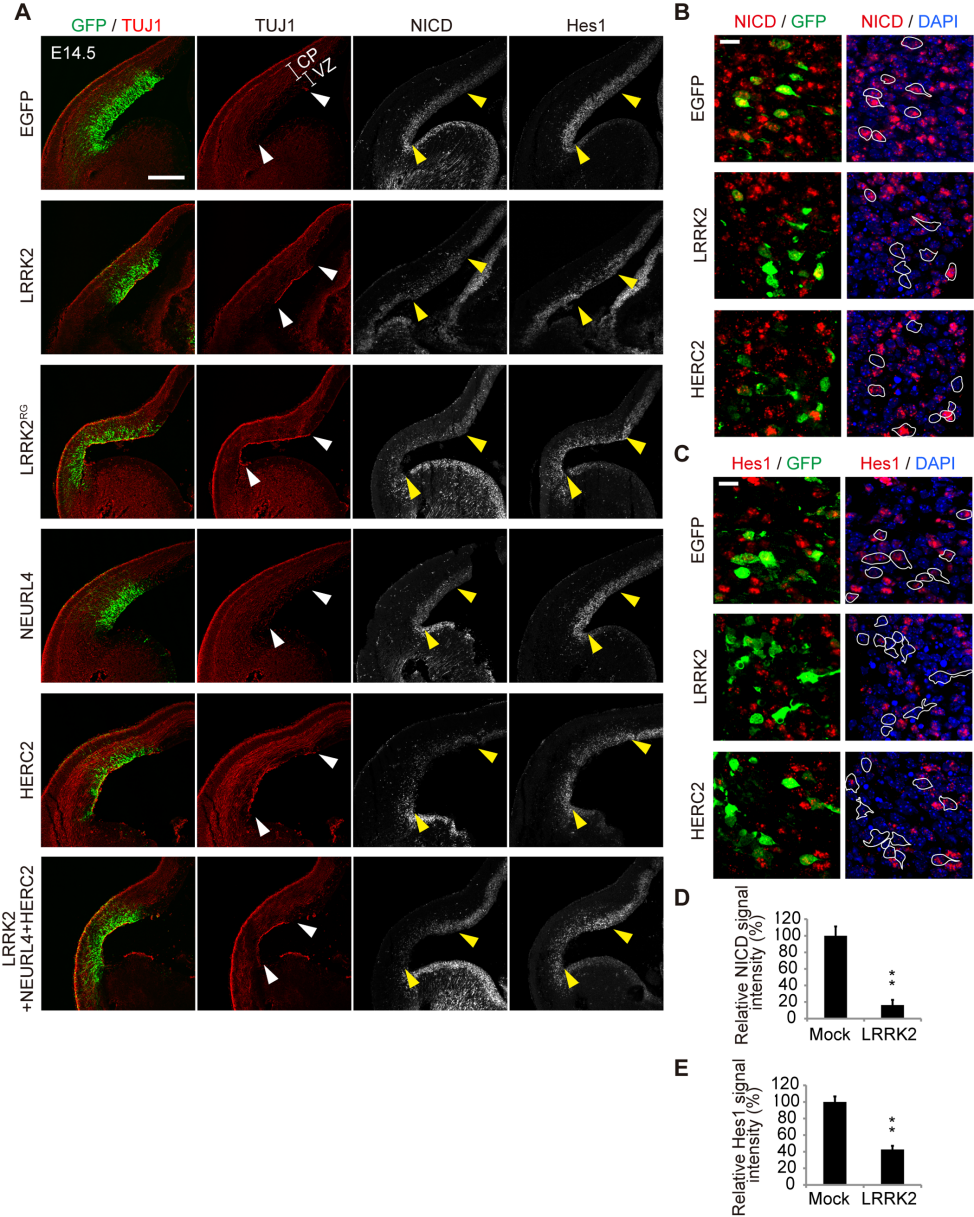
LRRK2 complex promotes neuronal differentiation *in vivo*. (A) Coronal sections of mouse dorsolateral telencephalon were immunostained for NICD, Hes1, neuron-specific class III β-tubulin (TUJ1) and GFP to stabilize the fates of cells transfected with the indicated genes 1 day after *in utero* electroporation at embryonic day 13.5 (E13.5). Empty vector pEF was used as a mock plasmid. EGFP was used as a reporter to visualize transfected cells. The regions of transgene expression are indicated by arrowheads. CP, the cortical plate and the intermediate zone; VZ, the ventricular and subventricular zones. Scale bar, 300 μm. (B, C) High-magnification images of samples from (A). Expression levels of NICD and Hes1 are suppressed in LRRK2- or HERC2-transfected cells. The boundaries of GFP-positive cells with intact nuclei are shown by white lines and are overlaid on the images stained for NICD or Hes1. Scale bars, 10 μm. (D, E) Immunoreactivity for NICD or Hes1 in EGFP-positive cells. Signal intensity was calculated from more than 200 cells from two animals in each group. **, *p* < 0.01 by Student’s *t*-test.

Given that the LRRK2 complex modulates Notch signaling by enhanced *cis*-inhibition, we next examined whether the expression of the LRRK2 complex affects neuronal differentiation in the development of the mouse brain, where the physiological Notch signal functions. The dorsolateral telencephalons of mouse embryos at embryonic day 13.5 (E13.5) were transfected with expression plasmids for LRRK2, NEURL4, HERC2, or a mixture of these plasmids via *in utero* electroporation and were then examined immunohistochemically 1 to 3 days post-transfection. TUJ1-positive differentiated neuronal cells emerged in the ventricular zones of the embryos transfected with LRRK2, HERC2 or the LRRK2/NEURL4/HERC2 mixture at 1 day post-transfection, and the immunoreactivity of NICD or Hes1 was decreased in areas of high transgene expression (Fig 6A). The appearance of TUJ1-positive cells was induced by ectopic HERC2 expression in WT embryos but not *LRRK2*-deficient embryos, suggesting that this phenomenon is LRRK2-dependent (S7A–S7C Fig). This observation suggests that Notch signaling is also suppressed by the LRRK2 complex in the mouse brain, leading to the accelerated neuronal differentiation of transfected cells. In contrast, the expression of NEURL4 alone failed to accelerate the neuronal differentiation of neural stem cells (Fig 6A), which is consistent with a result obtained in the reporter assay (Fig 4C).

Destruction of the normal ventricular zone structure or cell death by electroporation is unlikely to lead to the appearance of TUJ1-positive cells because NICD and Hes1 immunoreactivity were also decreased at E14.5 (i.e., 24 h post-transfection), when the overall structure of the ventricular zone was relatively preserved (Fig 6B–6E) and because the extent of cell death among LRRK2, HERC2 and NEURL4 transfectants was similar (S7D Fig).

At a later stage of development post-transfection with LRRK2, HERC2 or the mixture of the LRRK2 complex, the layer of cells positive for Pax6 (a marker for neuronal stem cells) in the ventricular zone was lost in the region of high transgene expression (S7E Fig). Conversely, ectopic TUJ1-positive cells were observed in the transfected regions (S7E Fig). Again, the transfection of NEURL4 alone did not have apparent effects (S7E Fig).

### Notch signaling regulates dopaminergic neuron survival

**Fig 7:**
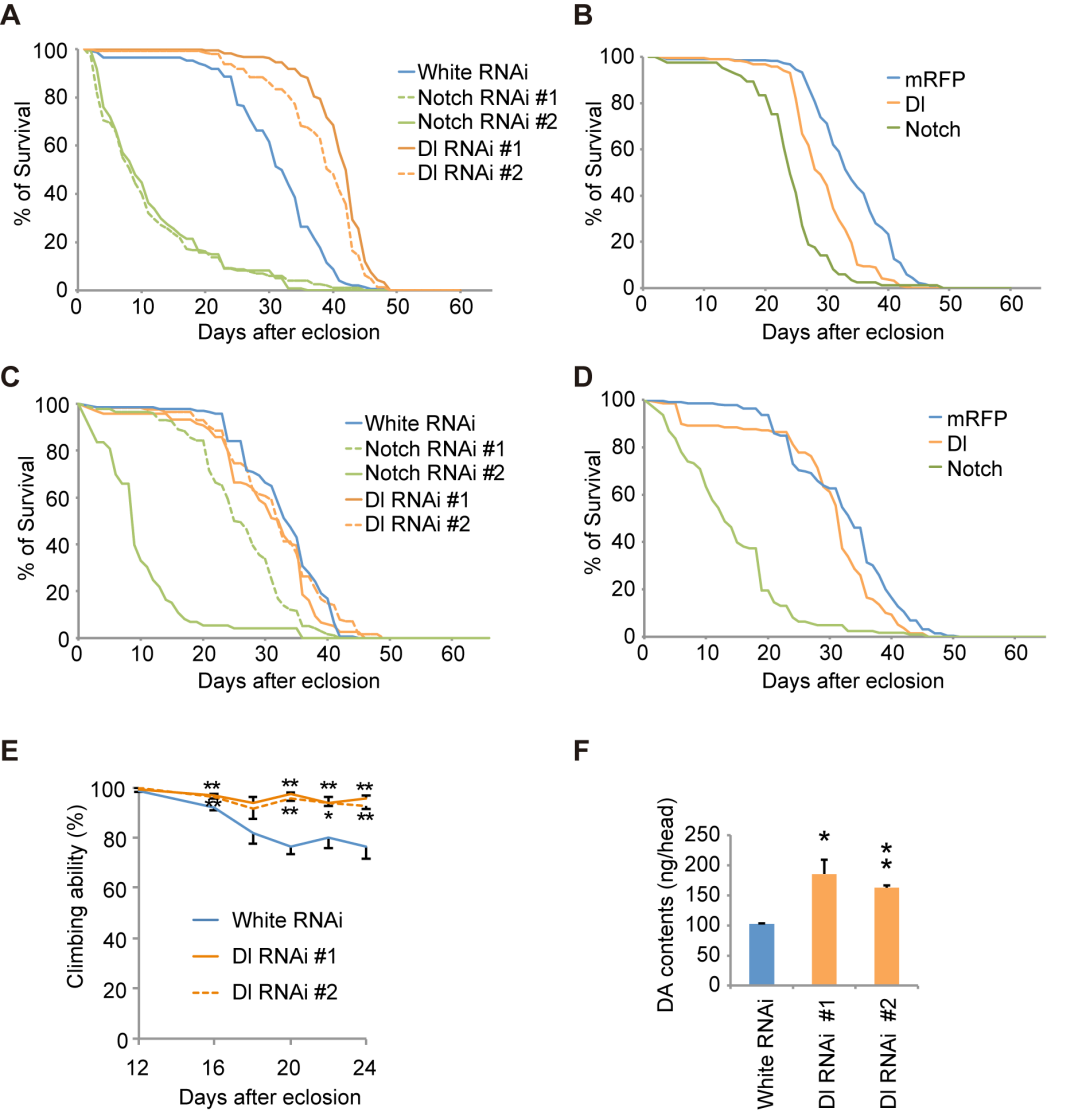
Notch signaling modulates the function of dopaminergic neurons. (A) Inhibition of Dl and Notch in the dopaminergic neurons during adulthood shortens and extends the lifespan, respectively, in *Drosophila*. *p* < 0.001 *vs*. control White RNAi, as determined by the log-rank test (n = 121–203). Flies harboring the indicated UAS-shRNAs were crossed with flies carrying tub-GAL80^ts^, TH-GAL4. Male offspring were raised at 18°C until eclosion, and the transgenes were expressed at 29°C during adulthood. (B) Ectopic expression of Dl and Notch in dopaminergic neurons during adulthood shortens the lifespan. *p* < 0.001 *vs*. mRFP, as determined by the log-rank test (n = 85–222). UAS-transgenes were expressed as in (A). (C) Inhibition of Dl in neurons other than dopaminergic neurons does not affect the lifespan, whereas Notch inhibition shortens the lifespan (*p* < 0.001, Notch RNAi #1 or #2 *vs*. White RNAi by the log-rank test. n = 78–178). Flies harboring the indicated UAS-shRNAs were crossed with flies carrying tub-GAL80^ts^, TH-GAL80, and elav-GAL4. Male offspring were raised at 18°C until eclosion, and the transgenes were expressed at 29°C during adulthood. (D) Ectopic expression of Dl in neurons other than dopaminergic neurons does not affect the lifespan, whereas ectopic Notch expression shortens the lifespan (*p* < 0.001 *vs*. mRFP by the log-rank test. n = 141–250). UAS-transgenes were expressed as in (C). (E) Age-dependent reduction of motor activity is improved by Dl inhibition. Flies were raised as in (A). (** *p* < 0.01, * *p* < 0.05 *vs*. White RNAi by one-way ANOVA). The data represent the mean ± SE from 2–6 trials. (F) Brain dopamine (DA) contents were increased by Dl inhibition (** *p* < 0.01, * *p* < 0.05 *vs*. White RNAi by one-way ANOVA. n = 3). Flies were raised as in (A), and adult male flies at 24 days of age were served.

**Fig 8:**
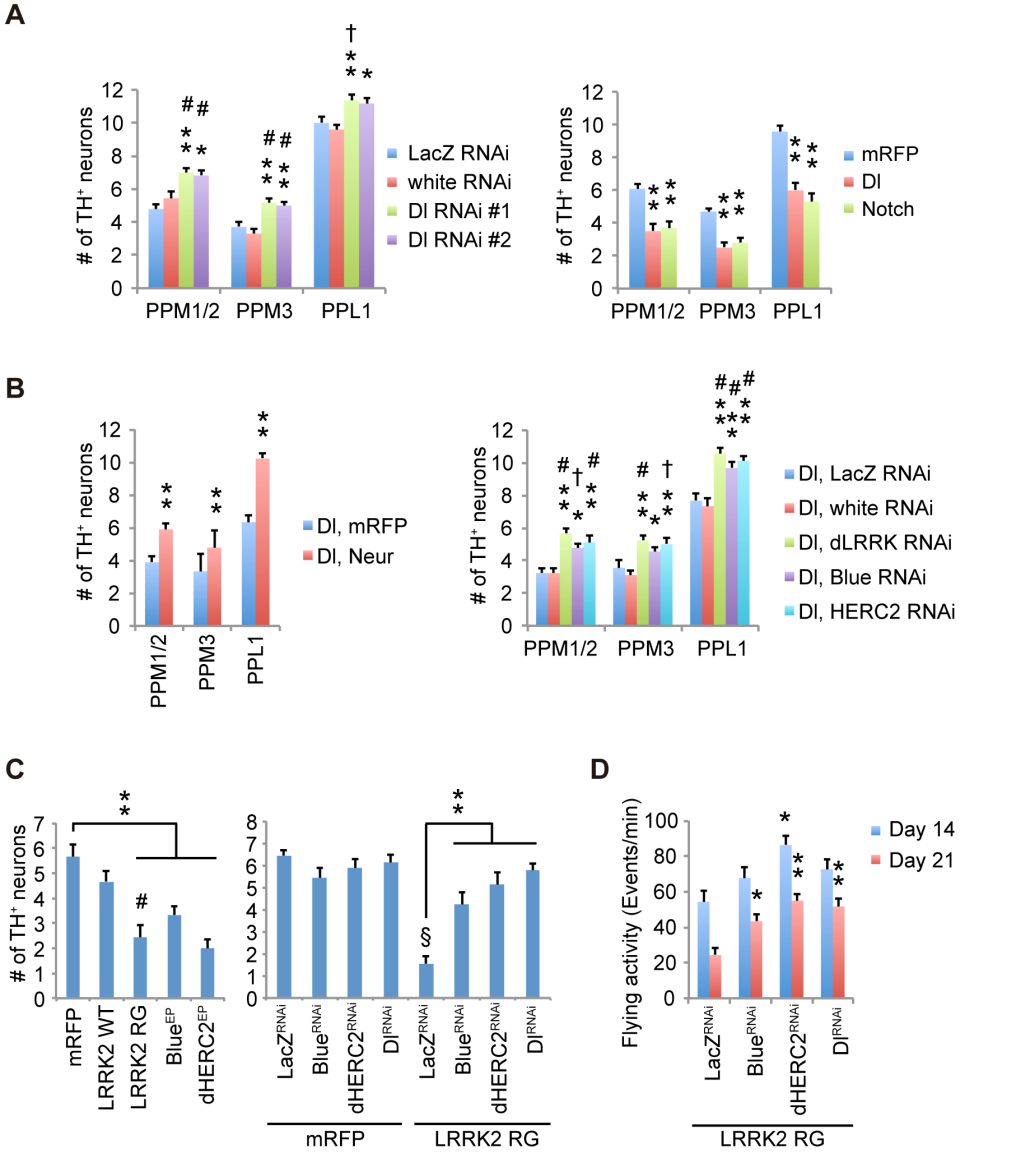
Notch signaling modulates the survival of dopaminergic neurons. (A) Survivability of dopaminergic neurons is improved by Dl inhibition (** *p* < 0.01, * *p* < 0.05 *vs*. White RNAi; # *p* < 0.01, † *p* < 0.05 *vs*. LacZ RNAi by one-way ANOVA. n = 10–11), whereas ectopic Dl and Notch expression led to neuronal loss (** *p* < 0.01 *vs*. mRFP by one-way ANOVA. n = 10). Flies were raised as in Fig 7A, and the dopaminergic neurons in the PPM1/2, PPM3 and PPL1 clusters of adult male flies at 21 days old were counted. (B) Enhanced Dl endocytosis by Neur overexpression protects the dopaminergic neurons from Dl overexpression-mediated neuronal loss (** *p* < 0.01 by Student’s *t*-test. n = 9). Suppression of dLRRK, Blue and dHERC2 also protects the neurons (** *p* < 0.01, * *p* < 0.05 *vs*. White RNAi; # *p* < 0.01, † *p* < 0.05 *vs*. LacZ RNAi by one-way ANOVA. n = 9). Flies were raised as in Fig 7A, and the dopaminergic neurons in the PPM1/2, PPM3 and PPL1 clusters of adult male flies at 21 days of age were counted. (C) LRRK2^RG^, Blue or dHERC2 overexpression leads to neuronal loss, whereas the suppression of Blue, dHERC2 and Dl rescues the loss of dopaminergic neurons caused by LRRK2^RG^. Flies were raised as in Fig 7A, and the dopaminergic neurons in the PPM1/2 cluster of adult male flies at 40 days of age were counted (Left graph, ** *p* < 0.01 *vs*. mRFP; # *p* < 0.01 *vs*. LRRK2 WT by one-way ANOVA. n = 9. Right, ** *p* < 0.01 *vs*. LacZ^RNAi^ with LRRK2 RG; § *p* < 0.01 *vs*. LacZ^RNAi^ with mRFP by one-way ANOVA. n = 9). (D) The flying activity of 14- and 21-day-old males expressing LRRK2^RG^ and the indicated shRNA constructs using the TH driver. Data are shown as the mean ± SE. ** *p* < 0.01, * *p* < 0.05 *vs*. LacZ RNAi. n = 10.

We next examined whether the modification of Notch signaling by the LRRK2 complex is associated with the dopaminergic neurodegeneration observed in PD. Canonical Notch signaling is activated and inhibited by ligands expressed in nearby cells and by ligands expressed autonomously within cells, respectively. We manipulated Notch and Dl expression in dopaminergic and non-dopaminergic neurons in adult flies. Dopaminergic-specific inactivation of Dl improved the survival and motor activity of adult flies, whereas ectopic expression of Dl in the dopaminergic neurons shortened the fly lifespan (Fig 7A, 7B and 7E). Dl inactivation or overexpression outside of the dopaminergic neurons had little effect on lifespan (Fig 7C and 7D). These results suggested that the changes to Notch signaling activity through *cis*-inhibition by Dl are important for the function of adult dopaminergic neurons. In contrast, the alterations of Notch levels in both dopaminergic and non-dopaminergic neurons had toxic effects (Fig 7A–7D). The suppression of Dl in dopaminergic neurons also increased the brain dopamine levels and improved dopaminergic neuron survival (Figs 7F, 8A and S8A). In contrast, both Dl and Notch overexpression in dopaminergic neurons decreased its survivability (Figs 8A and S8A), and the neuronal loss by Dl overexpression was suppressed by dLRRK, Blue or dHERC2 inhibition and Neur overexpression (Figs 8B, S3H, S3I and S8B).

LRRK2^RG^ exhibited a strong *cis*-inhibition activity (Fig 4D), and dopaminergic expression of LRRK2^RG^ led to dopaminergic neuronal loss in the PPM1/2 clusters, where neuronal excitation-dependent Notch activation could be detected (Figs 8C, S8C and S9). This neuronal loss and the reduction of flying behavior, which is associated with dopaminergic activity, were suppressed by Dl, Blue or dHERC2 inactivation (Fig 8C and 8D) [30]. In contrast, the overexpression of Blue or dHERC2 in dopaminergic neurons also caused neuronal loss (Fig 8C). Altogether, these results implied that the excess inhibition of Notch signaling affects the function and survival of adult dopaminergic neurons, and such inhibition could be mediated by the LRRK2 complex harboring ROC pathogenic mutations.

## Discussion

We identified two novel LRRK2-associated proteins, NEURL4 and HERC2, which link LRRK2 to the regulation of Dll1/Dl turnover through the endosomal pathway. The LRRK2/NEURL4/HERC2 complex slows the turnover of Dll1, which negatively regulates Notch signaling. This complex promotes the differentiation of neural stem cells during mouse development and modulates the function and survival of mature dopaminergic neurons in adult *Drosophila*.

### Roles of the LRRK2 complex in Dll1/Dl regulation

HERC2 belongs to the HERC family, which is characterized by the RCC1-like domain or RLD and HECT-type ubiquitin ligase domains. Another HERC family protein, HERC1, has been implicated in vesicle transport [31]. The RLD domains of HERC1 have a guanine-exchanging factor (GEF) activity for ARF1 and certain Rab proteins and can bind to the clathrin heavy chain, implying that HERC1 is involved in endocytosis and vesicular transport [31, 32]. NEURL4 belongs to the Neuralized family, a member of which is a RING-type ubiquitin ligase for Notch ligands [33]. Recently, it has been reported that HERC2 and NEURL4 regulate centrosome architecture [17]. dLRRK inactivation causes an abnormal centrosome phenotype in *Drosophila* cells, suggesting a functional link among LRRK2/dLRRK, HERC2 and NEURL4 [34].

Although the individual functions of LRRK2, HERC2 and NEURL4 remain to be fully elucidated, our results strongly suggest that the LRRK2 complex regulates the activity of Rab small GTPases. Based on the current results from fly genetic analysis and live-cell imaging, the LRRK2 complex can promote the endocytosis and recycling of Dll1 via early and recycling endosomes, thereby preventing Dll1 from trafficking to the late endosome and thus from lysosomal degradation (S10A Fig). The LRRK2 complex preferentially bound to Rab5 rather than Rab11 or Rab7, raising the possibility that the LRRK2 complex acts at the early endosomes to determine the fate of Dll1 (S2C–S2E Fig). The recycled Dll1 would presumably bind to Notch on the cell surface or at the endosomal compartments, thereby blocking Notch competency. HERC2 has the capacity to bind to Dll1, and NEURL4 appears to be a scaffold or adaptor protein that can form a four-part complex with LRRK2, HERC2 and Dll1. LRRK2 and NEURL4 appear to promote the ubiquitination of Dll1 by HERC2 (S10B and S10C Fig), which is likely to stimulate Dll1 endocytosis rather than degradation (Fig 5). Unlike Blue in *Drosophila*, overexpression of NEURL4 alone did not fully inhibit Notch signaling. This result might arise from a difference in molecular regulation between mammals and *Drosophila*. There is a good example of this situation in PINK1 and Parkin, two PD gene products. Overexpression of Parkin fully rescues PINK1 loss-of-function phenotypes in *Drosophila*, whereas Parkin cannot function in the absence of PINK1 in human cultured cells due to a more strict regulation of Parkin by PINK1 [35, 36]. Further studies will be required to understand the exact roles and regulatory mechanisms of LRRK2, NEURL4, HERC2 and other binding partners in the trafficking and regulation of Dll1.

### Roles of LRRK2 GTPase and kinase activities in Notch *cis*-inhibition

LRRK2 associates with NEURL4 through the ROC domain, and the GTPase activity appears to regulate its binding to NEURL4. The pathogenic LRRK2^RG^ mutant, which exhibits normal kinase activity but a modest impairment of GTP hydrolysis, increased the complex formation with NEURL4 and HERC2; LRRK2^TN^, which almost entirely lacks GTP hydrolysis and protein kinase activities, further enhanced the association compared with WT or LRRK2^RL^ with increased GTP hydrolysis activity [37]. The stable formation of the LRRK2 complex showed a good correlation with the intensity of Notch *cis*-inhibition. In contrast, a limited effect of LRRK2 kinase activity on *cis*-inhibition was observed under our experimental conditions, and we have no evidence that LRRK2 directly phosphorylates NEURL4 and HERC2 (S10D and S10E Fig). However, considering that the Neur∆RING wing phenotype with LRRK2^KD^ coexpression was milder than that with LRRK2^GS^ (S3D Fig), and that the Hes1 suppression effect by LRRK2^KD^ is slightly smaller than LRRK2^WT^ or LRRK2^GS^ (S4B Fig), we cannot exclude the possibility that there is a step that requires LRRK2 kinase activity in Dll1/Dl regulation.

LRRK1, the closest paralogue of LRRK2 in mammals, has both kinase activity-dependent and-independent functions to regulate the vesicular trafficking of the EGFR [38]. LRRK1 forms a complex with the EGFR in a kinase activity-independent manner, and its kinase activity regulates the transport of the EGFR from early to late endosomes. Thus, LRRK2 kinase activity may regulate the vesicular trafficking of Dll1 rather than the formation of the complex with Dll1. In this model, the kinase-dead LRRK2 will not transport Dll1 to either the cell surface or the lysosomes. The escaped Dll1 may bind to recycling Notch in the endosomal compartments, thereby suggesting that the total amount of Dll1, rather than the subcellular localization of Dll1, affects Notch *cis*-inhibition. The idea that LRRK2 has kinase-dependent and-independent functions might be supported by a comparative study of LRRK2 knockout mice and LRRK2 kinase-dead knock-in mice, where LRRK2 kinase-dead mice exhibit milder phenotypes than LRRK2 knockout mice or different phenotypes from LRRK2 knockout mice [6]. Another possibility is that endogenous LRRK2 may be recruited to the kinase-inactive LRRK2 complex, contributing to kinase-dependent regulation because LRRK2 can dimerize [39, 40]. Further molecular dissection will be required to address these possibilities.

### Potential contribution of Notch signaling to PD etiology

LRRK2 is highly expressed in the regions of high proliferative and migratory activity and cell differentiation or cell death, including the ventricular and subventricular zones of the embryonic telencephalon during neurogenesis [4]. However, LRRK2-knockout mice exhibit no developmental abnormalities [41], suggesting that many regulators of Notch and other signaling molecules achieve robust regulation during development. It has been reported that Notch activity is impaired in the adult brain of α-Synuclein transgenic mice and in mouse embryonic stem cells overexpressing α-Synuclein, raising the possibility that the pathogenic mechanisms of α-Synuclein and LRRK2 could converge with the dysfunction of the endosomal trafficking that compromises Notch signaling because α-Synuclein inhibits Rab-dependent endosomal trafficking [42, 43]. The retromer component Vps35 is another gene product for autosomal-dominant PD [44, 45]. The retromer regulates the vesicular sorting from the endosome to the trans-Golgi network or the plasma membrane, and a PD-associated mutation of Vps35 impairs these functions [46]. Because LRRK2 has been reported to genetically associate with Vps35, the PD-associated Vps35 mutation may also modulate Notch signaling [47].

The findings that Notch and Dll1 are substrates of the γ-secretase complex and that Notch signaling participates in learning and memory and microtubule dynamics imply a potential link between Notch signaling and Alzheimer’s disease (AD) pathogenesis. However, the involvement of Notch signaling in the neurodegeneration process remains largely uncharacterized. Our study has revealed that the conditional suppression of Dl in adult dopaminergic neurons unexpectedly improved the function and survival of dopaminergic neurons in *Drosophila*. However, manipulation of the Notch expression level (both increased and decreased) exhibited neurotoxicity pan-neuronally. A recent study has demonstrated that Notch activation in olfactory receptor neurons is dependent on both the neuronal response and Dl [14]. In this study, Notch activation dependent on dopaminergic excitation was also detected in a few dopaminergic neurons of the PPM2 and PPM3 clusters and in unidentified cells, which supports the idea that Notch signaling contributes to dopaminergic activity in mature neurons (S9 Fig).

Notch signaling also contributes to the regulation of synaptic plasticity, spine density and neuronal morphology in the adult brain. Conditional deletion of Notch1 in the postnatal mouse hippocampus has revealed that Notch signaling is essential for both long-term potentiation and long-term depression, along with learning and short-term memory in response to synaptic activity of the hippocampal neurons [48]. Synaptic plasticity is important for adaptive motor control and procedural memory in the striatum, and its impairment could account for the onset and progression of the motor symptoms of PD.

Neurogenesis is impaired in many transgenic mouse models for AD, although its relevance to AD pathogenesis remains controversial [49]. Impaired neurogenesis originates from Notch signal suppression in mice that express the AD-associated mutant Presenilin 1 [50]. Because the suppression of Notch signal intensity will decrease the population of neural stem cells in the brain, an attractive explanation for age-dependent neurodegeneration in *LRRK2*- or *α-synuclein*-linked PD is that impaired Notch signaling diminishes the maintenance of neuronal progenitor cells in the adult brain, making the brain more vulnerable to neuronal damage. The finding of impaired neurogenesis in LRRK2^GS^ transgenic mice appears to support this idea [51]; however, the existence of neurogenesis in the mature substantia nigra continues to be debated [52, 53].

In conclusion, this study demonstrated that LRRK2 and newly identified binding proteins regulate Notch activity by regulating the vesicular trafficking of Dll1/Dl. The alteration of Notch signaling in adult dopaminergic neurons in *Drosophila* modulates the function and survival of these cells, which may be associated with the neurodegeneration caused by LRRK2 mutations. Future studies addressing the involvement of LRRK2-Notch signaling pathways in the mammalian striatal dopaminergic system will contribute to an understanding of PD development.

## Materials and Methods

### Ethics statement

This study was approved by the Ethics Committee of Kyoto University, Japan (approval number: MedKyo14306). Carbon dioxide inhalation was used for a euthanasia method of mice. All surgical procedures were performed according to the rules set forth by the Ethics Committee of Kyoto University.

### Antibodies, plasmids and reagents

Western blot analysis using cultured cells, mouse brain tissue and *Drosophila* tissue samples was performed as described, using ECL prime solution (GE Healthcare) [30, 54]. The blot images were obtained with X-ray film or Image Quant LAS 4000 mini (GE Healthcare). All densitometry was performed using ImageJ software. Antibodies used in this study are described in S1 Text.

Reporter plasmid for Notch signaling activity using a Hes1 promoter (pHes1-luc) was generated in Kageyama’s lab. pCMV-Tag4-mouse Notch1-FLAG was a kind gift from Drs. K. Katsube and K. Sakamoto and subcloned into a pcDNA5/FRT vector. pMXs-rat Dll1-IG was a kind gift from Dr. Y. Goto, and the insert was sub-cloned into a pcDNA5/FRT vector. Rat Dll1 was PCR-amplified from pMXs-rat Dll-IG with new restriction enzyme cutting sites at both ends and with its stop codon deleted, and it was subcloned into a pcDNA3 vector with a C-terminal FLAG-6x His tag (pcDNA3-FLAG-His). For SNAP-Dll1, the SNAP-tag was PCR amplified from a pSNAPf vector (New England Biolabs) and inserted into the extracellular domain of rat Dll1 using In-Fusion cloning kit (Clontech). Plasmids for human LRRK2 have been reported elsewhere [9, 55] and were further sub-cloned into a pEF vector. Full-length cDNA of NEURL4 was obtained from Kazusa DNA Research Institute and sub-cloned into pcDNA3-FLAG-His or pEF. To clone full-length HERC2, total RNA was extracted from HEK293T cells, and cDNA was synthesized using a PrimeScript 1^st^ strand cDNA synthesis kit (Takara Bio). Partial clones were created by RT-PCR using PfuUltraII Fusion HS DNA polymerase (Agilent Technologies) and sequenced. Potential PCR errors were fixed by PCR mutagenesis to code the same amino acid sequences as the reference sequence (NP_004658.3). Full-length HERC2 cDNA was made by the ligation of the partial clones and cloned into pEGFP-C, pcDNA3 and pEF vectors. pEGFP-Rab7 and pEGFP-Rab11 plasmids were kindly provided by Dr. M. Fukuda. Other Rab members were cloned in pEGFP-C in our lab.

### Purification and identification of LRRK2-binding proteins

Flp-In 293 cells (Life Technologies) stably expressing FLAG-tagged human LRRK2 or parent cells were grown in suspension culture (Joklik-modified Eagle’s minimum essential medium with 5% (vol/vol) fetal bovine serum [FBS]). The cell pellet (2.4 x 10^8^ cells) was homogenized in lysis buffer (50 mM Tris pH 7.4, 120 mM NaCl, 5 mM EDTA, 10% (vol/vol) glycerol, 1% (vol/vol) Trion-X100) supplemented with Complete protease inhibitor cocktail (Roche Applied Science). The soluble fraction of the suspension was immunoprecipitated with anti-FLAG M2 affinity gel (Sigma-Aldrich) and washed five times in lysis buffer. The fractions eluted with 200 μg ml^-1^ 3x FLAG peptide (Sigma-Aldrich) were resolved using SDS-PAGE. Specific bands detected by silver staining were excised for in-gel digestion. The digest extracted from the gel was subjected to online HPLC-MS/MS, followed by informatics-based identification of the proteins (Nippon Proteomics).

### Cell lines

SH-SY5Y and HEK293T cells have been described previously [56, 57]. Mouse embryonic fibroblasts (MEFs) were isolated from *LRRK2-*deficient mice. HeLa cell line was purchased from Clontech. SH-SY5Y cells were cultured in Dulbecco’s modified Eagle’s medium (DMEM)/F-12 (Sigma-Aldrich) supplemented with non-essential amino acid (NEAA) and 10% (vol/vol) FBS. HEK293T, HeLa cells and MEFs were cultured in DMEM supplemented with NEAA and 10% FBS. CHO cells were cultured in nutrient mixture F-12 (Sigma-Aldrich) supplemented with NEAA and 10% FBS. CHO and HeLa cell lines stably expressing transgenes were generated with the Flp-In system following the manufacturer’s instructions (Life Technologies). Briefly, pFRT/lacZeo plasmid was transfected into CHO or HeLa cells using Lipofectamine LTX (Life Technologies), and Zeocin (Life Technologies) was added to culture medium to select stably transfected cells. Zeocin-resistant cells were picked up and checked for single-site integrations of pFRT/lacZeo into the cell genomes by Southern blot. Among the cells with pFRT/lacZeo single-site integration, the cells with the highest β-galactosidase activity were chosen for further use. To produce individual stable cell lines, the genes of interest were subcloned into pcDNA5/FRT. pcDNA5/FRT plasmids containing the genes of interest were co-transfected with pOG44, and the cells stably expressing the genes of interest were selected with hygromycin. To select and maintain the stability of transfected cells, 300 μg ml^-1^ hygromycin or 250 μg ml^-1^ Zeocin for CHO cells and 300 μg ml^-1^ hygromycin or 150 μg ml^-1^ Zeocin for HeLa cells were used. MEFs stably expressing SNAP-Dll1 with EGFP-Rab5, EGFP-Rab7 or EGFP-Rab11 were produced by retroviral infection with pMX-puro harboring the transgenes and subsequent puromycin selection. For transient transfection, plasmids and siRNA duplexes (Silencer and Stealth, Life Technologies) were transfected using Lipofectamine 2000 (Life Technologies) and Lipofectamine RNAiMAX (Life Technologies), respectively, according to the manufacturer’s instructions.

### Co-immunoprecipitation assays

The indicated plasmids were transfected into HEK293T cells using Lipofectamine 2000 (Life Technologies). Forty-eight hours after transfection, the cells were lysed with RIPA buffer (50 mM Tris pH 8.0, 150 mM NaCl, 1% (vol/vol) NP-40, 0.5% (wt/vol) sodium deoxycholate, 0.1% (wt/vol) SDS), which was supplemented with Complete cocktail. For immunoprecipitation of the FLAG-tagged proteins, cell lysate was added to anti-FLAG gel. For immunoprecipitation of other proteins, specific antibodies were added to the cell or tissue lysates and precipitated with Protein G affinity gel (GE Healthcare). Following incubation at 4°C for 3 h, the gels were washed five times with RIPA buffer. The proteins immunoprecipitated with anti-FLAG gel were eluted with 200 μg ml^-1^ 3x FLAG peptide, and the proteins immunoprecipitated with antibodies other than the anti-FLAG antibody were eluted in SDS-PAGE sample buffer. The eluted proteins were analyzed by SDS-PAGE followed by Western blotting.

### Notch reporter assay

SH-SY5Y cells were used as ‘Notch cells’ and co-cultured with CHO cells stably transfected with rat Dll1-HA (Dll1-CHO cells), which were used as ‘Ligand cells’. SH-SY5Y cells were plated in 24-well plates the day prior to transfection. A 20%-confluent monolayer of cells was co-transfected with a reporter plasmid that encoded firefly luciferase in conjunction with the *Hes1* promoter (pHes1-Luc) and a plasmid for *Renilla* luciferase (pGL4-TK-Rluc, Promega) to monitor the transfection efficiency, together with an empty plasmid or plasmids for the LRRK2 complex. Dll1-CHO cells (5 x 10^4^) were added to each well of SH-SY5Y cells 12 h after transfection and co-cultured for an additional 36 h. The co-cultured cells were lysed and subjected to the reporter assay using a dual-luciferase reporter assay system (Promega) and Fluoroskan Ascent FL (ThermoFisher) following the manufacturers’ instructions.

### Live cell imaging and image analyses

HeLa cells stably expressing Dll1 with a SNAP-tag in the extracellular region on an 8-well Lab-Tek chamber slide were labeled with 5 μM SNAP-Surface Alexa Fluor 647 for 20 min at 37°C; the cells were subsequently washed three times with normal medium without phenol red. The cell chamber was placed in a CO^2^- and temperature-controlled microscope incubator and immediately imaged using a laser-scanning microscope system (LSM780/LSM7 Live, Carl Zeiss) with a 63x/1.2 water-immersion objective. Image stacks of multiple areas were taken every 15 min at 1.8-μm intervals. The Dll1 fluorescence intensity at the cytoplasmic membrane was measured by subtracting the Dll1 intensity at cellular vesicles from the total Dll1 intensity using the Surface tool in Imaris software (Bitplane). The quantifications of colocalized Dll1 with EGFP-Rabs and the quantifications of NICD and Hes1 in EGFP-positive cells were analyzed using the Coloc tool in Imaris software. *LRRK2*-deficient MEFs stably expressing Dll1-SNAP and Rab-EGFP were transiently transfected with LRRK2 or LacZ twice sequentially using Lipofectamine LTX, which achieved ~95% transfection efficiency estimated by EGFP transfection in parallel. Imaging analysis was performed as in HeLa cells.

### *Drosophila* genetics

Fly culture and crosses were performed on standard fly food containing yeast, cornmeal and molasses, and flies were raised at 25°C unless otherwise indicated. To generate *UAS-hLRRK2* mutant transgenic lines, the cDNA for *hLRRK2* ^*RG*^, *hLRRK2* ^*GS*^ *and hLRRK2* ^*KD*^ from a pcDNA3.1 vector was subcloned into the *pUAST* vector. The introduction of transgenes into the *Drosophila* germline and the establishment of transgenic lines into a *w* ^*−*^background were performed by BestGene Inc. (Chino Hills, CA). *UAS-dHERC2* RNAi, *UAS-Blue* RNAi, *UAS-dLRRK* RNAi, *UAS-Dl* RNAi (#1, v37288; #2, v37287 [58]) and *UAS-Notch* RNAi (#1, v27229; #2, v1112 [59]) were obtained from the Vienna *Drosophila* RNAi Center. *Dl* ^*3*^ was obtained from the Kyoto Stock Center. *Notch-LexAVP16* and *LexOP-dGFP* were kind gifts of Drs. Lieber and Struhl [14]. *UAS-Shi* ^*ts1*^ was a kind gift of Dr. Kitamoto. All other fly stocks and *GAL4* lines used in this study were obtained from the Bloomington *Drosophila* Stock Center and have been previously described: *UAS-dLRRK* [9]; *e03680* as a *dLRRK* null allele [9]; *UAS-hLRRK2* [60]; and *hs-Dl* [61]. Full details of *Drosophila* genotypes used in this study are described in S2 Text.

### Generation of a *Blue* mutant allele

The *p{EPgy2}EY12221* insertion line (*blue* ^*EP*^ line) from the Bloomington *Drosophila* Stock Center was mobilized using ∆*2–3* transposase. We screened for imprecise excision of the *p{EPgy2}EY12221* by genomic PCR and identified a new insertion of an additional 11 bp of a genomic fragment containing 7 bp of the 5’ part of the *Blue* gene and 78 bp of a sequence derived from *p{EPgy2}* (named *blue* ^*47b-2*^).

### Dl endocytosis assay

The internalization assay of anti-Dl was performed basically following the reported procedures using *hs-Dl* crosses at 26°C [27, 33]. Briefly, third instar larvae wing imaginal discs were dissected in *Drosophila* Schneider’s medium containing 10% FBS and were incubated in the same medium at RT for 15 min with mouse anti-Delta antibody (C594.9B, Developmental Studies Hybridoma Bank, 1:100), which recognises the extracellular domain of Dl. The wing discs were washed three times for 15 min in Schneider’s medium with 10% FBS, fixed in 4% paraformaldehyde/PBS, washed three times for 15 min in 0.1% Triton X-100/PBS and visualized with a second antibody (anti-mouse Alexa 647, Life Technologies, 1:200). Dpp areas of the wing pouch were imaged using a laser-scanning microscope system (SP5, Leica Microsystems) with a 63x/1.40NA oil-immersion objective. Z-Stack images consisting of 80–100 slices were taken at a step size of 0.5-μm. Reconstituted confocal slices 5 to 8 μm below the apical surface were used for colocalization analysis of Dl with Rab7-GFP or Rab11-GFP, in which GFP and anti-Dl signals were outlined using Photoshop. Overlapped and non-overlapped signals over 0.06 μm^2^ were extracted using the analyze particles tool in ImageJ.

### *Drosophila* lifespan assay, and climbing and flying assays

Approximately 20 adult flies per vial were maintained at 29°C, transferred to fresh fly food and scored for survival every 2 days. To control for isogeny, the fly lines were generated in the *w* ^*-*^ wild-type genetic background or were backcrossed to the *w* ^*-*^ background for six generations to match the genetic background. Climbing and flying assays were performed as previously described [9, 62].

### Dopamine measurement

Dopamine measurement of the fly brain tissues was performed as previously described [9].

### *in utero* electroporation and immunohistochemistry

*in utero* electroporation and immunohistochemistry were performed as previously described [63]. The embryos were sacrificed at 1 or 3 days after electroporation. The brains were excised, fixed in 4% (wt/vol) paraformaldehyde, embedded in OCT compound and sectioned at 16 μm. To detect NICD and Hes1, a TSA Plus Cyanine 3 System (Perkin-Elmer) was used for signal amplification.

### Generation of *LRRK2*-deficient mice

A floxed Neo selection cassette was inserted at the end of exon 15 of murine *LRRK2* gene by homologous recombination in TT2 ES cells, which were derived from F1 hybrid of C57BL/6 and CBA mice [64]. The homologous recombination events in the ES cells were confirmed by Southern blot with the 5′ and 3′ external probes and the neo probe. The ES cell clones were then injected into ICR 8-cell stage embryos. The chimeric offspring were crossed to C57BL/6J mice to obtain heterozygous mutant mice. To remove the neo gene and generate a frame-shift (LRRK2 KO) allele, mutant mice were crossed with CAG-Cre mice that express Cre recombinase in mature oocytes [65]. The mutant mice were subsequently backcrossed onto C57BL/6J background for seven generations, which were then intercrossed to obtain homozygous LRRK2 KO mice. Although the expression of an N-terminal fragment of LRRK2 (1–558 aa) with an additional 97 amino acids from the frame-shift allele was envisaged, we confirmed that no signals were detected at the expected migration position in Western blot analysis with an anti-LRRK2 antibody that recognized the N-terminal site of LRRK2. Genotyping of *LRRK2 KO* mice (Accession No. CDB0609K: http://www.cdb.riken.jp/arg/mutant%20mice%20list.html) was performed by PCR using following primer set (forward, 5’-TGCATACCCAGACATTGAATTATGTTTTACC-3’; reverse, 5’-GGTCCTGATGTTTTGTGCAGG-3’) and WT and mutant alleles were amplified as a 335-bp and a 275-bp DNA fragments, respectively. The *LRRK2*-deficeint MEF used here was obtained from this mouse line. Detailed information will be provided upon request.

### Statistical analysis

Data are expressed in mean values ± SEM. To compare quantitative data of multiple groups, repeated measures one-way ANOVA was performed. *p* < 0.05 was considered as significant and if a statistically significant difference was detected by one-way ANOVA, the Bonferroni multiple comparison test was done as a *post hoc* test. Statistical significance of difference between two groups was determined by the two-tailed unpaired Student’s *t*-test.

## Supporting Information

S1 Fig Domain analysis of LRRK2, NEURL4 and HERC2. (A-E) Specificity of anti-NERUL4 and anti-HERC2 antibodies. HeLa cells transfected with mock (A,A’,D), NEURL4 (B,B’) or HERC2 (E) siRNA were immunostained with anti-NEURL4 (green), anti-HERC2 (green) and anti-Rab7 (red). (C,C’) HeLa cells transfected with Myc-NEURL4 were immunostained with anti-Myc (green) and anti-Rab7 (red). Scale bars, 10 μm (A-C’) and 50 μm (D, E). (F,G) NEURL4 binds to LRRK2 through the ROC domain of LRRK2. HEK293T cell lysate transfected with plasmids for a series of truncated LRRK2 constructs with FLAG-tag as depicted in (I) or a mock plasmid, with or without a plasmid for Myc-NEURL4, was subjected to immunoprecipitation with anti-FLAG antibody and analyzed by Western blotting with the indicated antibodies. (H) Identification of the NEURL4 interaction domains with LRRK2 and HERC2. NEURL4 binds to LRRK2 and HERC2 via NHR3-4 and NHR5-6, respectively. (I) Schematic of LRRK2 domain structure and its truncated mutants. LRR, Leucine-rich repeats; ROC, Ras of complex proteins domain; COR, C-terminal of ROC domain; kinase, kinase domain; WD, WD40 repeats. (J) Schematic of NEURL4 domain structure and its truncated mutants.
(TIF)

S2 Fig NEURL4 and Neur bind to Dll1. (A) NEURL4 binds to Neur in HEK293T cells. Note that Neur signals in cell lysate were not detected under this condition. (B) NEURL4 does not compete with Neur for binding to Dll1. The asterisk indicates non-specific bands that appeared with anti-HA (clone 12CA5). (C-E) FLAG-LRRK2, FLAG-NEURL4 and FLAG-HERC2 were co-expressed with a series of Rab GTPases with an EGFP tag in HEK293T cells. Co-immunoprecipitated Rabs with anti-FLAG antibody were detected with anti-GFP antibody.
(TIF)

S3 Fig Expression and genetic analyses for *dLRRK*, *Blue* and *dHERC2* in fly lines. (A) The levels of *blue* transcript were measured using quantitative RT-PCR (qRT-PCR), which was then normalized by housekeeping *rp49* levels. Expression of Blue was induced by the ubiquitous *daughterless* (*Da*)-*GAL4* driver. The GAL4 expression caused a 30-fold increase of the *blue* transcripts in the *p{EPgy2}EY12221* (*Blue* ^*EP*^) line. (B) The levels of Blue protein in *Da-GAL4*, *Da*> *Blue* ^*EP*^, *Da*> *Blue* ^*RNAi*^ and heterozygous *Blue* ^*47b-2*^ flies were examined using Western blotting. Tubulin signal served as a loading control. (C) The levels of *dHERC2* transcript were increased by ~6-fold in the *EP* ^*G17171*^ line (*dHERC2* ^*EP*^, Bloomington 33296) in the presence of *GAL4*. (D) The lack of endogenous dLRRK partially rescues the wing margin defects. Transgenes were driven by the *Dpp-GAL4* as in Fig 3B. (E) Co-expression of Neur and dHERC2 by *Dpp-GAL4* minimally affects the wing margin formation, whereas the wing size is reduced. Total wing and Dpp areas (highlighted in green) of the indicated genotypes were graphed. **, *p* < 0.01; *, *p* < 0.05 by one-way ANOVA. (F) The LRRK2 complex does not modulate the *Serrate* phenotype. Ectopic wing margin bristles produced by Serrate overexpression are indicated (arrowheads). (G) The LRRK2 complex does not modulate the *Notch* mutant phenotype. *N* ^*55e11*^ flies exhibit additional wing vein formation (white dashed circle) and thickening of the wing veins (black dashed circle). The manipulation of LRRK2 complex activity did not affect them. (H) The levels of *dHERC2* transcript in the *dHERC2 RNAi* fly were estimated using qRT-PCR as in (A). (I) The levels of dLRRK protein in *Da-GAL4* crosses expressing LacZ RNAi, dLRRK RNAi (v22139 and v22140) and dLRRK (dLRRK OE) were examined using Western blotting with anti-dLRRK. The actin signal served as a loading control. The asterisk indicates non-specific bands.
(TIF)

S4 Fig Reconstitution of non cell-autonomous Notch signaling in cultured cells. (A) SH-SY5Y cells were transfected with Hes1 reporter plasmid along with control (LacZ) or Notch1 expression plasmids. CHO cells stably expressing Dll1 (D) and parental CHO (P) cells were co-cultured as signal-sending and mock cells, respectively. Notch signal intensity assessed by the Hes1 promoter assay is shown as the relative Hes1 promoter activity. (B) LRRK2 kinase activity does not contribute to the suppressive potency of Notch signaling. ***, *p* < 0.001; **, *p* < 0.01 *vs*. LacZ by one-way ANOVA.
(TIF)

S5 Fig LRRK2, NEURL4 and HERC2 do not affect Notch1 turnover. (A) Expression of LRRK2, NEURL4, or a combination of LRRK2 and NEURL4 does not stabilize Dll1. Turnover of Dll1 was analyzed as in Fig 5A and 5B. The level of Dll1 remaining at different time points was graphed. Data are shown as the mean ± SE from three repeated experiments. (B) Effects of HERC2 overexpression on Dll1 turnover. Graph represents the mean ± SE from three repeated experiments. (C) The levels of endogenous Notch1 (Left graph, immature p300 form) and (Right, mature p120 form) in the cells of Fig 5A at different time points were plotted as the percentage of initial Notch1 level (0 hour of CHX treatment). Data are shown as the mean ± SE from four repeated experiments. (D) Knockdown efficiency of a mixture of siRNA against LRRK2, NEURL4 and HERC2 (siLNH) was confirmed by Western blotting. (Left) Endogenous LRRK2 was immunoprecipitated with anti-dFoxO (Control IP) or anti-LRRK2 (LRRK2 IP) antibodies from HEK293 cell lysate. HEK293 cell lysate that expressed FLAG-LRRK2 was used as a positive control. (Right) Endogenous NEURL4 and HERC2 signals were detected in HEK293 cells, and these signals were abolished by siLNH treatment. (E) Dynamics of cell surface Dll1. HEK293 cells stably expressing Dll1-SNAP were transfected with control siRNA duplex (Control RNAi) or siLNH (LNH RNAi). Cell surface Dll1 was labeled with SNAP-Surface Alexa Fluor 647 for 20 min at 37°C. Note that there was a tendency of Dll1 signal reduction by the LRRK2 complex knockdown. Representative gray-scale cell images are shown 0–6 h after the washout of the tracking dye (t = 0). (F) Inactivation of LRRK2 complex decreases the amount of cell surface Dll1. Data are presented as the mean ± SE for six independent experiments, with 15–24 cells counted per sample. **, *p* < 0.01 *vs*. Mock RNAi at 6 hr by Student’s *t*-test.
(TIF)

S6 Fig Live-cell imaging analysis of Dll1 turnover. (A) LRRK2 and LacZ expression in LRRK2^-/-^ MEFs stably expressing Dll1-SNAP along with EGFP-Rab5, EGFP-Rab7 or EGFP-Rab11, which were used in live-cell imaging analysis in Figs 5E and S6B. (B) Representative live images (green, EGFP-Rab; red, Dll1-SNAP; white, colocalized signals) of Fig 5E at 0 and 2 h post labeling are shown.
(TIF)

S7 Fig Endogenous LRRK2 activity is required for neuronal differentiation by ectopic expression of HERC2. (A) Targeting strategy for generation of *LRRK2 KO* mice. The locations of the 5′ and 3′ external probes used for Southern blot are indicated. Sites of genotyping PCR primers are also shown (see also Materials and Methods). Restriction sites for Southern blot: B, BamHI; E, EcoNI. Numbers, the exon numbers of the *LRRK2* gene. (B) LRRK2 expression in the striatum of LRRK2 WT and KO mice was analyzed by Western blotting using anti-LRRK2 antibody. (C) Coronal sections of *LRRK2* ^*+/+*^ and *LRRK2* ^*-/-*^ mouse littermate embryos were immunostained with TUJ1 and anti-GFP 24 h after *in utero* electroporation with the indicated genes together with EGFP at E13.5 as in Fig 6A. The regions of transgene expression are indicated by arrowheads. (D) Effects of transient expression of LRRK2, NEURL4 or HERC2 on cell death in the developmental mouse brain. Serial sections of the mouse dorsolateral telencephalon shown in Fig 6A were immunostained with anti-single stranded DNA (ssDNA) and counterstained with methyl green for nuclei to estimate the numbers of dead cells. (E) Coronal sections of the dorsolateral telencephalon were immunostained with TUJ1 or anti-Pax6 3 days after *in utero* electroporation of the indicated genes at E13.5. Images represent typical examples that were reproducibly observed from at least two independent embryos in multiple experiments. The regions of transgene expression are indicated by arrowheads. CP, the cortical plate; IZ, the intermediate zone; VZ; the ventricular and subventricular zones. Scale bar, 300 μm.
(TIF)

S8 Fig Morphology of the dopaminergic neurons in flies analyzed in Fig 8. (A) Flies were raised as in Fig 8A, and the dopaminergic neurons in the PPM1/2, PPM3 and PPL1 clusters of adult male flies at 21 days of age were visualized with anti-TH antibody. Scale bar, 20 μm. (B) Flies were raised as in Fig 8B, and 21-day-old flies were stained as in (A). (C) Flies were raised as in Fig 8C, and 40-day-old flies were stained as in (A).
(TIF)

S9 Fig Excitation-dependent Notch activation in dopaminergic neurons. (A) PPL3 (arrowheads) and some PPM3 (circles) dopaminergic neurons exhibit Notch activation after TrpA1-dependent excitation. PPL3 is characterized as a TH-GAL4-responding, anti-TH-negative neuron [66]. To determine whether Notch signaling is activated in TH-positive neurons by neuronal excitation, Notch reporter (N-LV, LexOP-dGFP [14]) flies harboring the tub-GAL80^ts^; TH-GAL4 driver and UAS-mCD8::mRFP; UAS-TrpA1 transgenes were raised at 18°C until 3–5 days post eclosion to suppress the TrpA1 expression; the flies were then shifted to 30°C for 3–4 days to permit TrpA1 and CD8::mRFP expression in TH-GAL4-responding neurons. Neurons were visualized with the indicated signals (dGFP and CD8::mRFP) or anti-TH staining. TrpA1 is a cation channel activated at 30°C, and tub-GAL80^ts^ is a temperature-sensitive GAL80 repressor against GAL4, the expression of which is regulated by the ubiquitous *tubulin* promoter [67]. (B) Higher magnification images of (A). PPL3 and PPM3 neurons positive for Notch activity were co-stained with anti-*elav*. Red and yellow arrowheads indicate the anti-elav signals of PPM3 and PPL3, respectively. (C) Some PPM2 dopaminergic neurons also exhibit excitation-dependent Notch activation. Notch reporter flies harboring the *tub-GAL80* ^*ts*^, *TH-GAL4* driver and *UAS-TrpA1* transgene or Notch reporter flies harboring the *TH-GAL4* driver and *UAS-shibire* ^*ts1*^ (*shi* ^*ts1*^) transgene were raised at 18°C until 3–5 days post eclosion and then shifted to 30°C for 3–4 days to permit transgene expression as in (A). *shi* ^*ts1*^ is a dominant-negative form of dynamin that blocks synaptic transmission at 30°C [67, 68]. We did not detect silencing-dependent Notch activation in the PPM2 or PPM3 neurons; however, some uncharacterized cells and neurites were GFP-positive independent of TH neuronal activity. Scale bars, 50 μm (A,C), 20 μm (B).
(TIF)

S10 Fig A model of Dl/Dll1 regulation by the LRRK2 complex. (A) LRRK2 complex stimulates endocytosis and recycling of Dl/Dll1. Increased activity of the LRRK2 complex or gain-of-function effects by the PD-associated mutation R1441G lead to the accumulation of Dl/Dll1, whereas loss-of-function of the LRRK2 complex results in Dl/Dll1 degradation via the late endosome-lysosome pathway. (B,C) The LRRK2 complex promotes Dll1 ubiquitination. HEK293T cell lysate transfected with the indicated cDNA and/or siRNA was subjected to immunoprecipitation with anti-FLAG antibody and analyzed by Western blotting with the indicated antibodies. The total amount of transfected cDNA was adjusted with vector DNA. ** *p* < 0.01 *vs*. Dll1-FLAG alone by one-way ANOVA, * *p* < 0.05 by Student’s *t*-test. Ub, ubiquitin. (D) FLAG-tagged LRRK2, HERC2 and NEURL4 were immunopurified using anti-FLAG beads and FLAG peptide. The amounts and purity were evaluated by CBB staining (arrowheads). (E) *In vitro* kinase assay of LRRK2. The indicated combination of proteins in the presence or absence of 2 mM GTP was added to kinase buffer containing recombinant GST-FoxO1-C as a LRRK2 substrate. Kinase assay by autoradiography was performed as described previously [55].
(TIF)

S1 Text Antibodies used in this study. (DOCX)

S2 Text Fly genotypes used in this study. (DOCX)
