# Supplementary figures and images for: The Parkinson’s Disease-Associated Protein Kinase LRRK2 Modulates Notch Signaling through the Endosomal Pathway
Source: PLoS Genet. 2015 Sep 10;11(9):e1005503. doi: 10.1371/journal.pgen.1005503 (PMC4565672; doi:10.1371/journal.pgen.1005503)

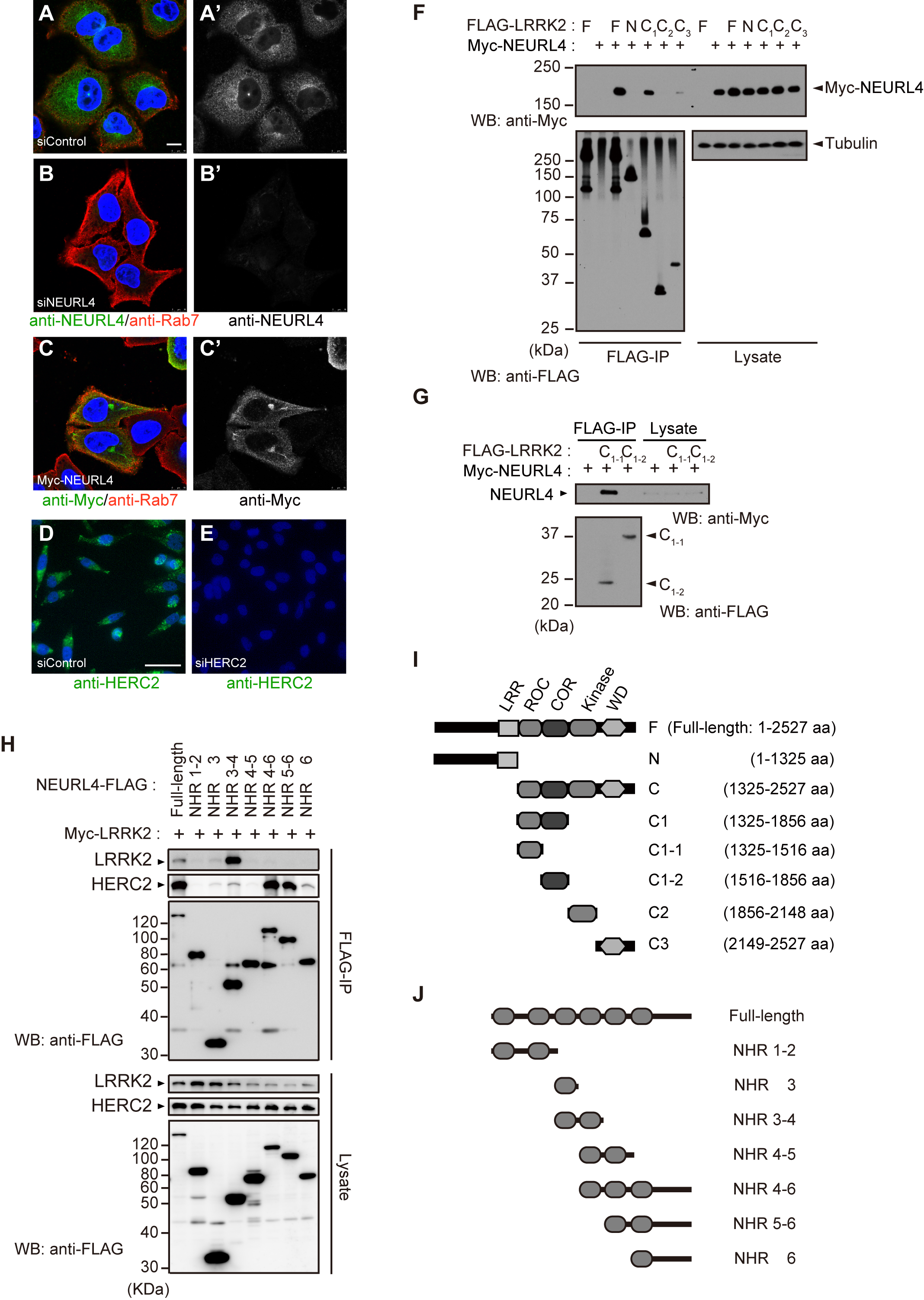

Supplement: S1 Fig — (A-E) Specificity of anti-NERUL4 and anti-HERC2 antibodies. HeLa cells transfected with mock (A,A’,D), NEURL4 (B,B’) or HERC2 (E) siRNA were immunostained with anti-NEURL4 (green), anti-HERC2 (green) and anti-Rab7 (red). (C,C’) HeLa cells transfected with Myc-NEURL4 were immunostained with anti-Myc (green) and anti-Rab7 (red). Scale bars, 10 μm (A-C’) and 50 μm (D, E). (F,G) NEURL4 binds to LRRK2 through the ROC domain of LRRK2. HEK293T cell lysate transfected with plasmids for a series of truncated LRRK2 constructs with FLAG-tag as depicted in (I) or a mock plasmid, with or without a plasmid for Myc-NEURL4, was subjected to immunoprecipitation with anti-FLAG antibody and analyzed by Western blotting with the indicated antibodies. (H) Identification of the NEURL4 interaction domains with LRRK2 and HERC2. NEURL4 binds to LRRK2 and HERC2 via NHR3-4 and NHR5-6, respectively. (I) Schematic of LRRK2 domain structure and its truncated mutants. LRR, Leucine-rich repeats; ROC, Ras of complex proteins domain; COR, C-terminal of ROC domain; kinase, kinase domain; WD, WD40 repeats. (J) Schematic of NEURL4 domain structure and its truncated mutants. (TIF) [file pgen.1005503.s001.tif]

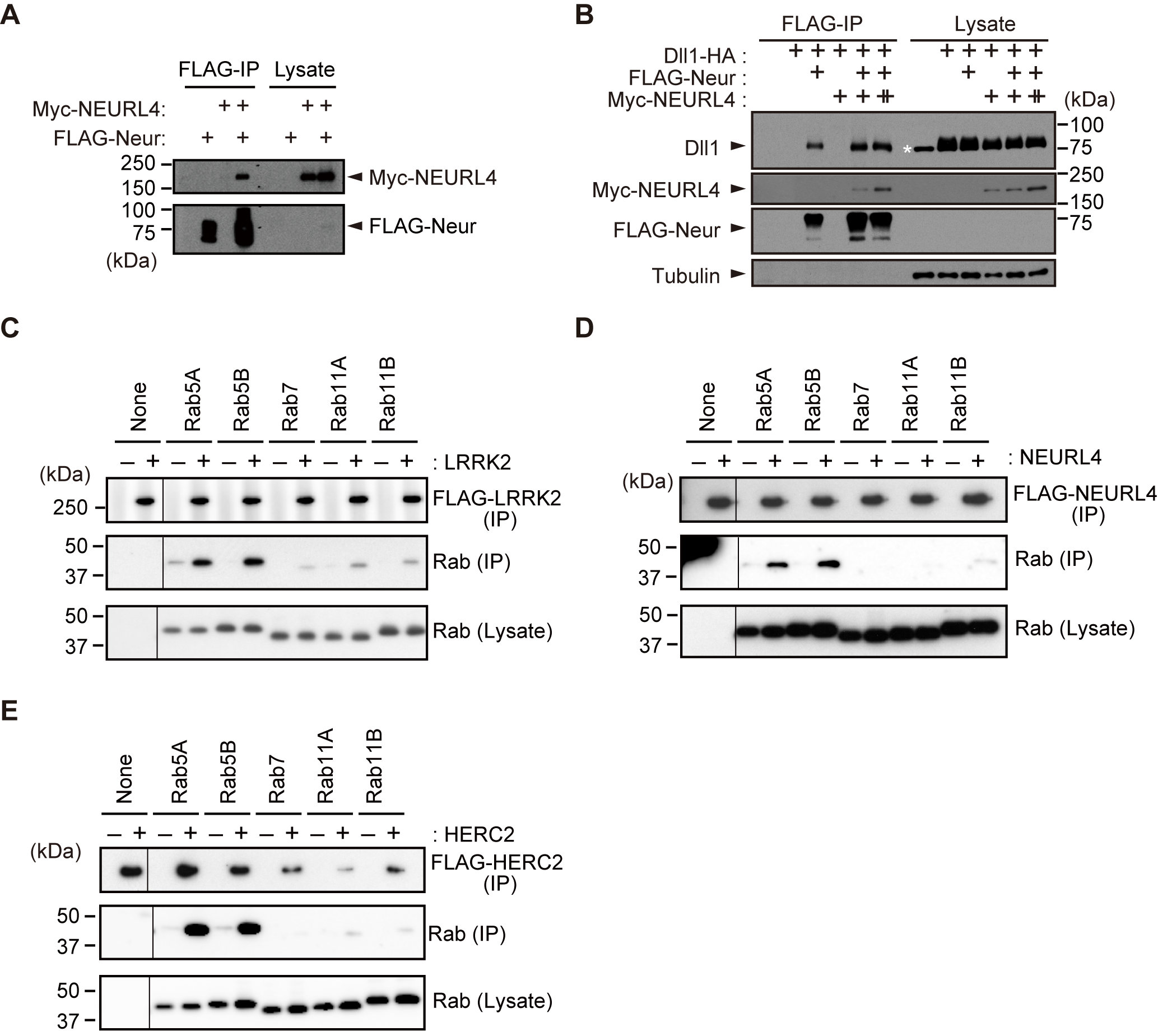

Supplement: S2 Fig — (A) NEURL4 binds to Neur in HEK293T cells. Note that Neur signals in cell lysate were not detected under this condition. (B) NEURL4 does not compete with Neur for binding to Dll1. The asterisk indicates non-specific bands that appeared with anti-HA (clone 12CA5). (C-E) FLAG-LRRK2, FLAG-NEURL4 and FLAG-HERC2 were co-expressed with a series of Rab GTPases with an EGFP tag in HEK293T cells. Co-immunoprecipitated Rabs with anti-FLAG antibody were detected with anti-GFP antibody. (TIF) [file pgen.1005503.s002.tif]

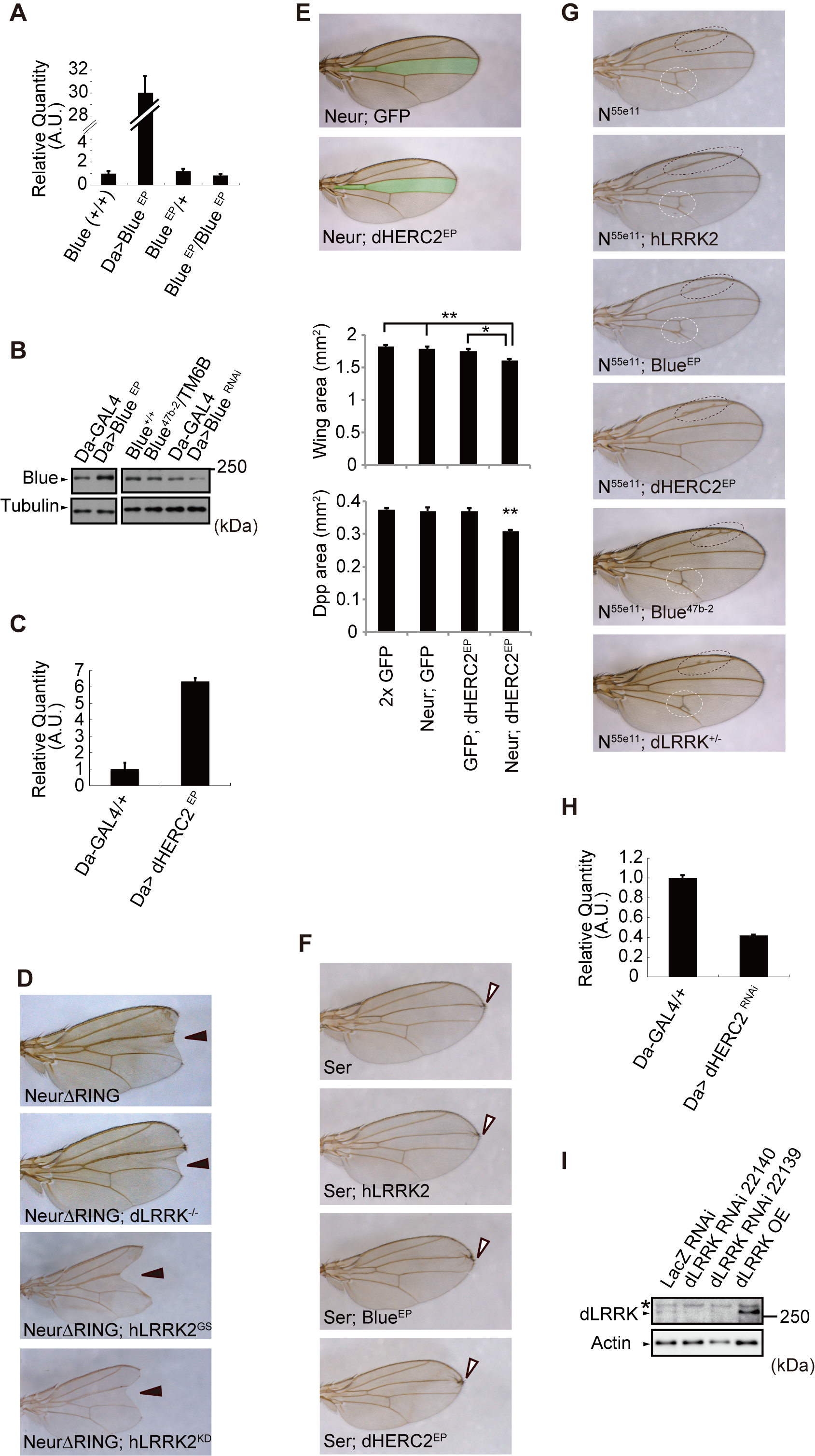

Supplement: S3 Fig — (A) The levels of blue transcript were measured using quantitative RT-PCR (qRT-PCR), which was then normalized by housekeeping rp49 levels. Expression of Blue was induced by the ubiquitous daughterless (Da)-GAL4 driver. The GAL4 expression caused a 30-fold increase of the blue transcripts in the p{EPgy2}EY12221 (Blue EP) line. (B) The levels of Blue protein in Da-GAL4, Da> Blue EP, Da> Blue RNAi and heterozygous Blue 47b-2 flies were examined using Western blotting. Tubulin signal served as a loading control. (C) The levels of dHERC2 transcript were increased by ~6-fold in the EP G17171 line (dHERC2 EP, Bloomington 33296) in the presence of GAL4. (D) The lack of endogenous dLRRK partially rescues the wing margin defects. Transgenes were driven by the Dpp-GAL4 as in Fig 3B. (E) Co-expression of Neur and dHERC2 by Dpp-GAL4 minimally affects the wing margin formation, whereas the wing size is reduced. Total wing and Dpp areas (highlighted in green) of the indicated genotypes were graphed. **, p < 0.01; *, p < 0.05 by one-way ANOVA. (F) The LRRK2 complex does not modulate the Serrate phenotype. Ectopic wing margin bristles produced by Serrate overexpression are indicated (arrowheads). (G) The LRRK2 complex does not modulate the Notch mutant phenotype. N 55e11 flies exhibit additional wing vein formation (white dashed circle) and thickening of the wing veins (black dashed circle). The manipulation of LRRK2 complex activity did not affect them. (H) The levels of dHERC2 transcript in the dHERC2 RNAi fly were estimated using qRT-PCR as in (A). (I) The levels of dLRRK protein in Da-GAL4 crosses expressing LacZ RNAi, dLRRK RNAi (v22139 and v22140) and dLRRK (dLRRK OE) were examined using Western blotting with anti-dLRRK. The actin signal served as a loading control. The asterisk indicates non-specific bands. (TIF) [file pgen.1005503.s003.tif]

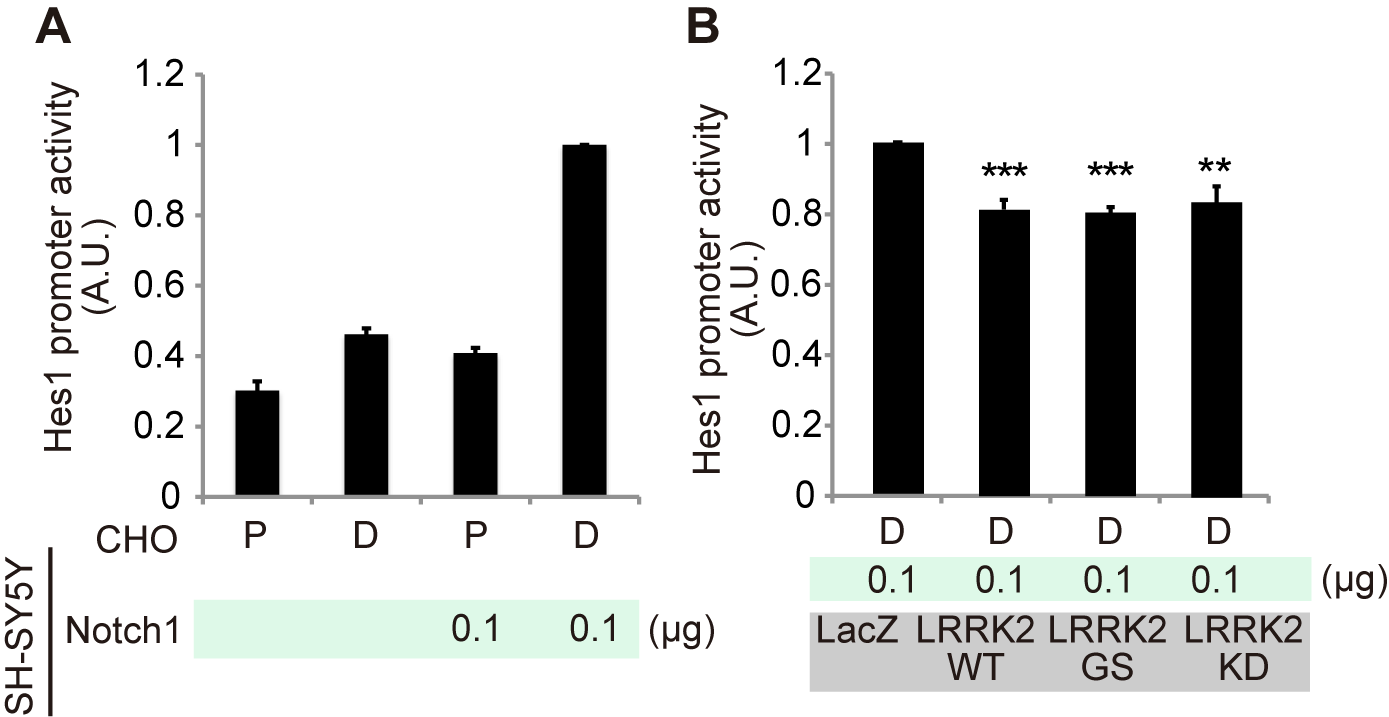

Supplement: S4 Fig — (A) SH-SY5Y cells were transfected with Hes1 reporter plasmid along with control (LacZ) or Notch1 expression plasmids. CHO cells stably expressing Dll1 (D) and parental CHO (P) cells were co-cultured as signal-sending and mock cells, respectively. Notch signal intensity assessed by the Hes1 promoter assay is shown as the relative Hes1 promoter activity. (B) LRRK2 kinase activity does not contribute to the suppressive potency of Notch signaling. ***, p < 0.001; **, p < 0.01 vs. LacZ by one-way ANOVA. (TIF) [file pgen.1005503.s004.tif]

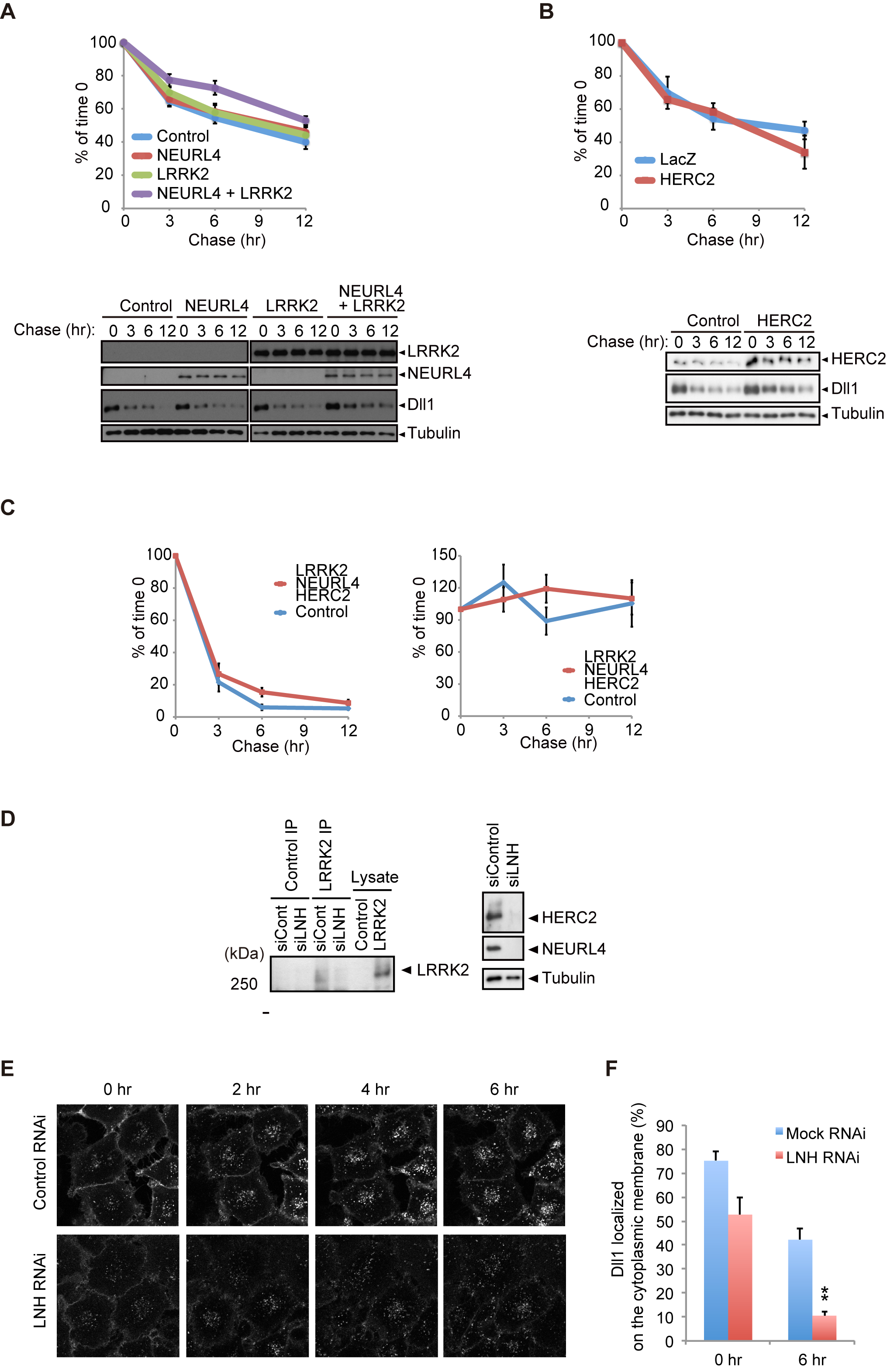

Supplement: S5 Fig — (A) Expression of LRRK2, NEURL4, or a combination of LRRK2 and NEURL4 does not stabilize Dll1. Turnover of Dll1 was analyzed as in Fig 5A and 5B. The level of Dll1 remaining at different time points was graphed. Data are shown as the mean ± SE from three repeated experiments. (B) Effects of HERC2 overexpression on Dll1 turnover. Graph represents the mean ± SE from three repeated experiments. (C) The levels of endogenous Notch1 (Left graph, immature p300 form) and (Right, mature p120 form) in the cells of Fig 5A at different time points were plotted as the percentage of initial Notch1 level (0 hour of CHX treatment). Data are shown as the mean ± SE from four repeated experiments. (D) Knockdown efficiency of a mixture of siRNA against LRRK2, NEURL4 and HERC2 (siLNH) was confirmed by Western blotting. (Left) Endogenous LRRK2 was immunoprecipitated with anti-dFoxO (Control IP) or anti-LRRK2 (LRRK2 IP) antibodies from HEK293 cell lysate. HEK293 cell lysate that expressed FLAG-LRRK2 was used as a positive control. (Right) Endogenous NEURL4 and HERC2 signals were detected in HEK293 cells, and these signals were abolished by siLNH treatment. (E) Dynamics of cell surface Dll1. HEK293 cells stably expressing Dll1-SNAP were transfected with control siRNA duplex (Control RNAi) or siLNH (LNH RNAi). Cell surface Dll1 was labeled with SNAP-Surface Alexa Fluor 647 for 20 min at 37°C. Note that there was a tendency of Dll1 signal reduction by the LRRK2 complex knockdown. Representative gray-scale cell images are shown 0–6 h after the washout of the tracking dye (t = 0). (F) Inactivation of LRRK2 complex decreases the amount of cell surface Dll1. Data are presented as the mean ± SE for six independent experiments, with 15–24 cells counted per sample. **, p < 0.01 vs. Mock RNAi at 6 hr by Student’s t-test. (TIF) [file pgen.1005503.s005.tif]

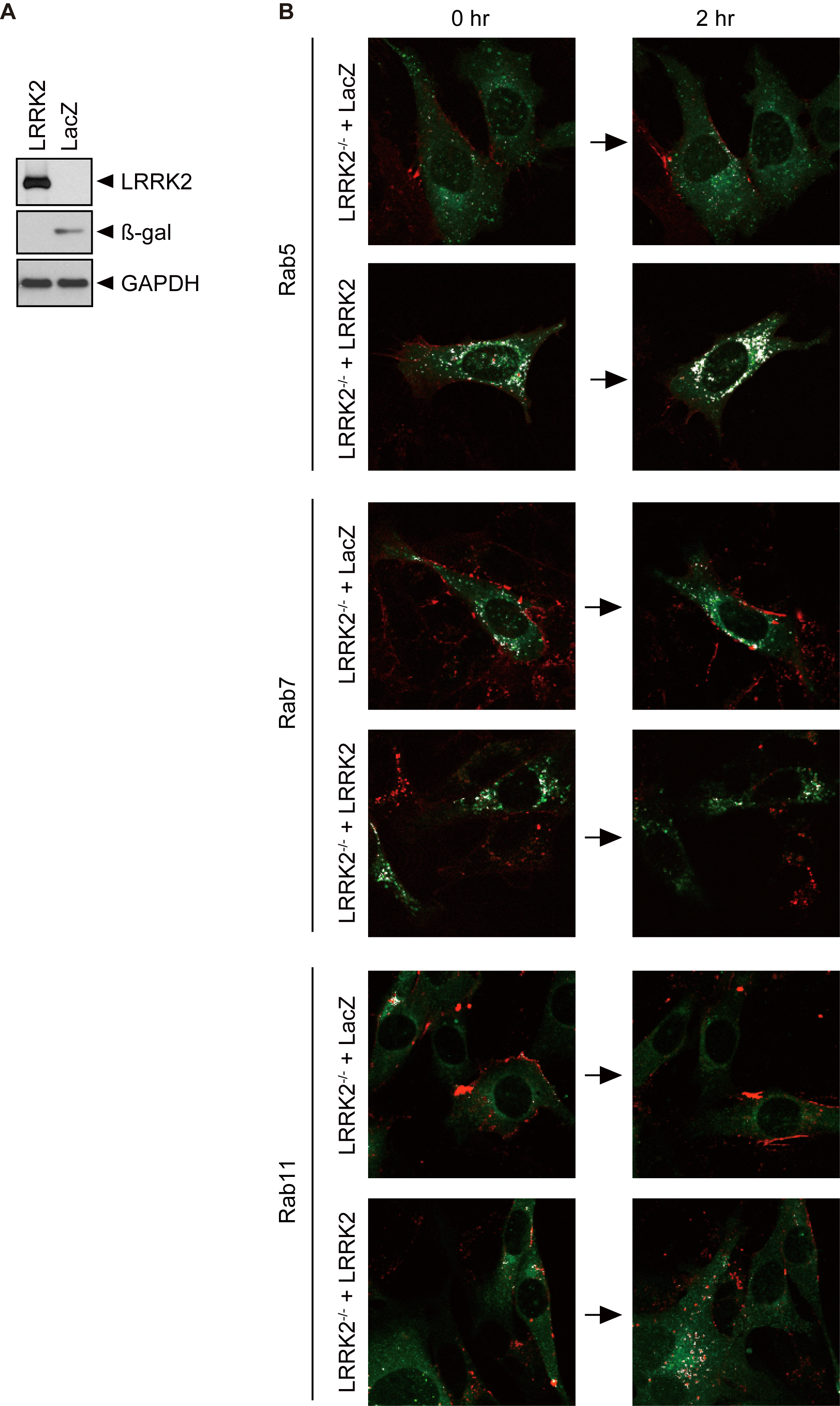

Supplement: S6 Fig — (A) LRRK2 and LacZ expression in LRRK2-/- MEFs stably expressing Dll1-SNAP along with EGFP-Rab5, EGFP-Rab7 or EGFP-Rab11, which were used in live-cell imaging analysis in Figs 5E and S6B. (B) Representative live images (green, EGFP-Rab; red, Dll1-SNAP; white, colocalized signals) of Fig 5E at 0 and 2 h post labeling are shown. (TIF) [file pgen.1005503.s006.tif]

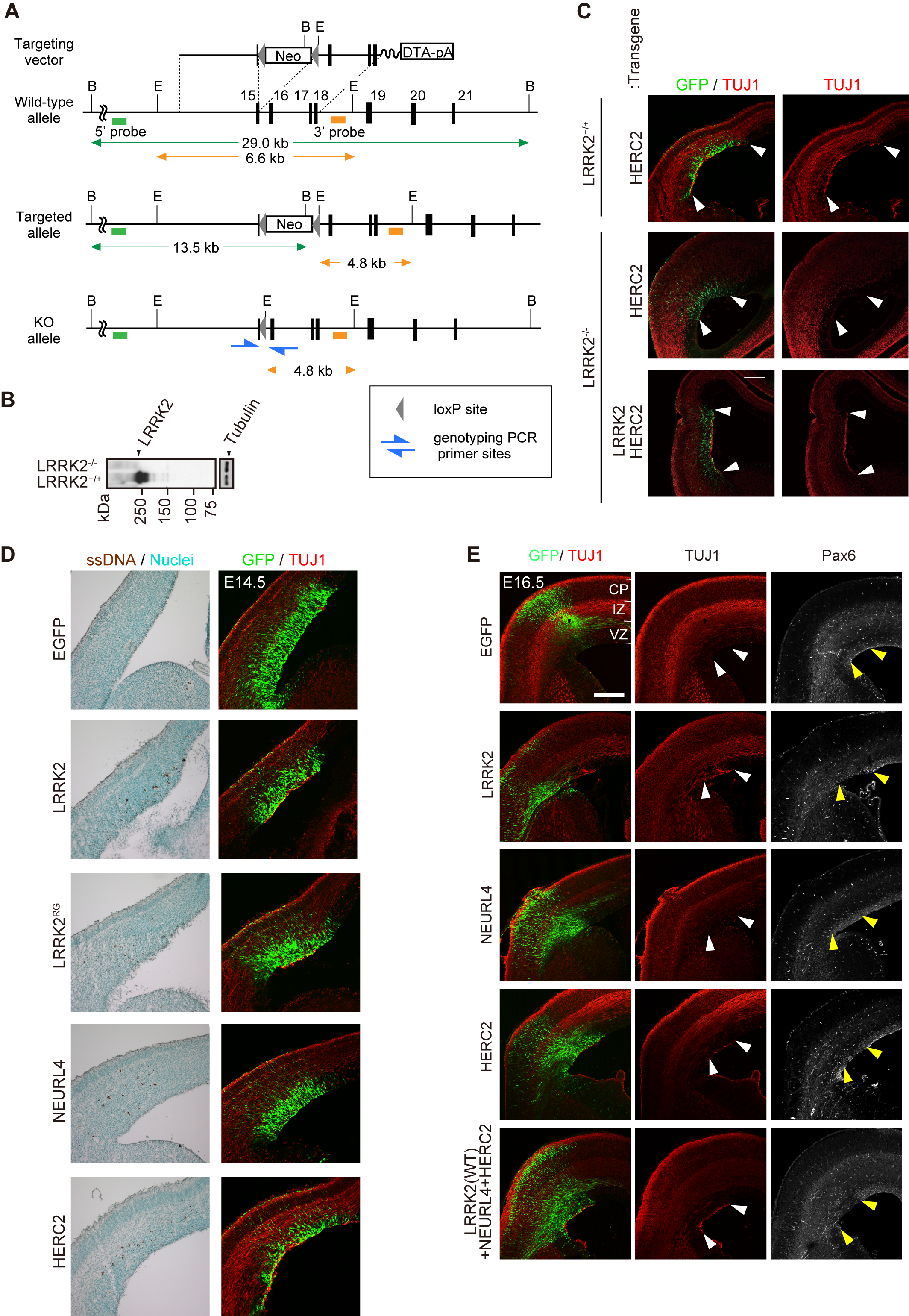

Supplement: S7 Fig — (A) Targeting strategy for generation of LRRK2 KO mice. The locations of the 5′ and 3′ external probes used for Southern blot are indicated. Sites of genotyping PCR primers are also shown (see also Materials and Methods). Restriction sites for Southern blot: B, BamHI; E, EcoNI. Numbers, the exon numbers of the LRRK2 gene. (B) LRRK2 expression in the striatum of LRRK2 WT and KO mice was analyzed by Western blotting using anti-LRRK2 antibody. (C) Coronal sections of LRRK2 +/+ and LRRK2 -/- mouse littermate embryos were immunostained with TUJ1 and anti-GFP 24 h after in utero electroporation with the indicated genes together with EGFP at E13.5 as in Fig 6A. The regions of transgene expression are indicated by arrowheads. (D) Effects of transient expression of LRRK2, NEURL4 or HERC2 on cell death in the developmental mouse brain. Serial sections of the mouse dorsolateral telencephalon shown in Fig 6A were immunostained with anti-single stranded DNA (ssDNA) and counterstained with methyl green for nuclei to estimate the numbers of dead cells. (E) Coronal sections of the dorsolateral telencephalon were immunostained with TUJ1 or anti-Pax6 3 days after in utero electroporation of the indicated genes at E13.5. Images represent typical examples that were reproducibly observed from at least two independent embryos in multiple experiments. The regions of transgene expression are indicated by arrowheads. CP, the cortical plate; IZ, the intermediate zone; VZ; the ventricular and subventricular zones. Scale bar, 300 μm. (TIF) [file pgen.1005503.s007.tif]

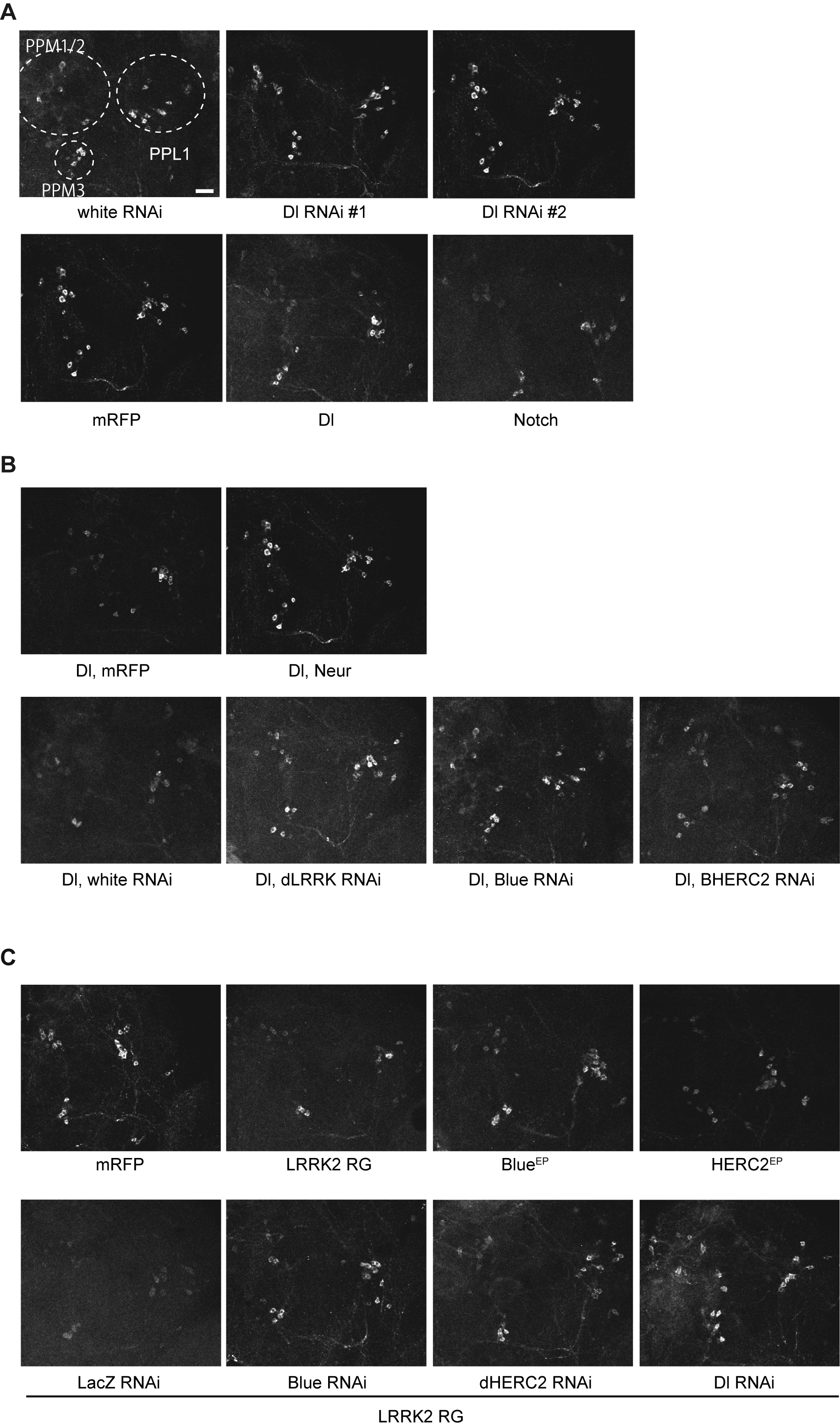

Supplement: S8 Fig — (A) Flies were raised as in Fig 8A, and the dopaminergic neurons in the PPM1/2, PPM3 and PPL1 clusters of adult male flies at 21 days of age were visualized with anti-TH antibody. Scale bar, 20 μm. (B) Flies were raised as in Fig 8B, and 21-day-old flies were stained as in (A). (C) Flies were raised as in Fig 8C, and 40-day-old flies were stained as in (A). (TIF) [file pgen.1005503.s008.tif]

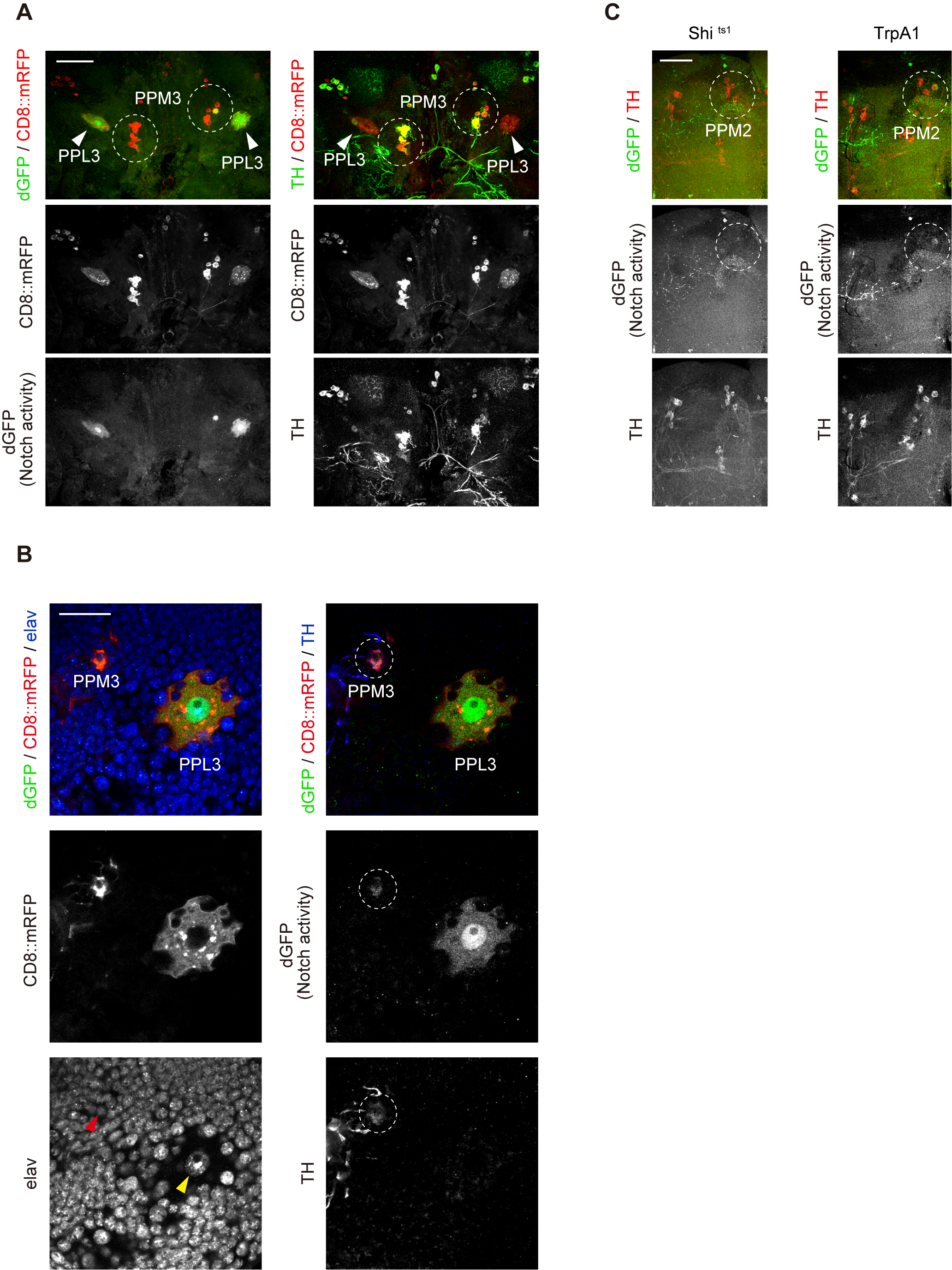

Supplement: S9 Fig — (A) PPL3 (arrowheads) and some PPM3 (circles) dopaminergic neurons exhibit Notch activation after TrpA1-dependent excitation. PPL3 is characterized as a TH-GAL4-responding, anti-TH-negative neuron [66]. To determine whether Notch signaling is activated in TH-positive neurons by neuronal excitation, Notch reporter (N-LV, LexOP-dGFP [14]) flies harboring the tub-GAL80ts; TH-GAL4 driver and UAS-mCD8::mRFP; UAS-TrpA1 transgenes were raised at 18°C until 3–5 days post eclosion to suppress the TrpA1 expression; the flies were then shifted to 30°C for 3–4 days to permit TrpA1 and CD8::mRFP expression in TH-GAL4-responding neurons. Neurons were visualized with the indicated signals (dGFP and CD8::mRFP) or anti-TH staining. TrpA1 is a cation channel activated at 30°C, and tub-GAL80ts is a temperature-sensitive GAL80 repressor against GAL4, the expression of which is regulated by the ubiquitous tubulin promoter [67]. (B) Higher magnification images of (A). PPL3 and PPM3 neurons positive for Notch activity were co-stained with anti-elav. Red and yellow arrowheads indicate the anti-elav signals of PPM3 and PPL3, respectively. (C) Some PPM2 dopaminergic neurons also exhibit excitation-dependent Notch activation. Notch reporter flies harboring the tub-GAL80 ts, TH-GAL4 driver and UAS-TrpA1 transgene or Notch reporter flies harboring the TH-GAL4 driver and UAS-shibire ts1 (shi ts1) transgene were raised at 18°C until 3–5 days post eclosion and then shifted to 30°C for 3–4 days to permit transgene expression as in (A). shi ts1 is a dominant-negative form of dynamin that blocks synaptic transmission at 30°C [67, 68]. We did not detect silencing-dependent Notch activation in the PPM2 or PPM3 neurons; however, some uncharacterized cells and neurites were GFP-positive independent of TH neuronal activity. Scale bars, 50 μm (A,C), 20 μm (B). (TIF) [file pgen.1005503.s009.tif]

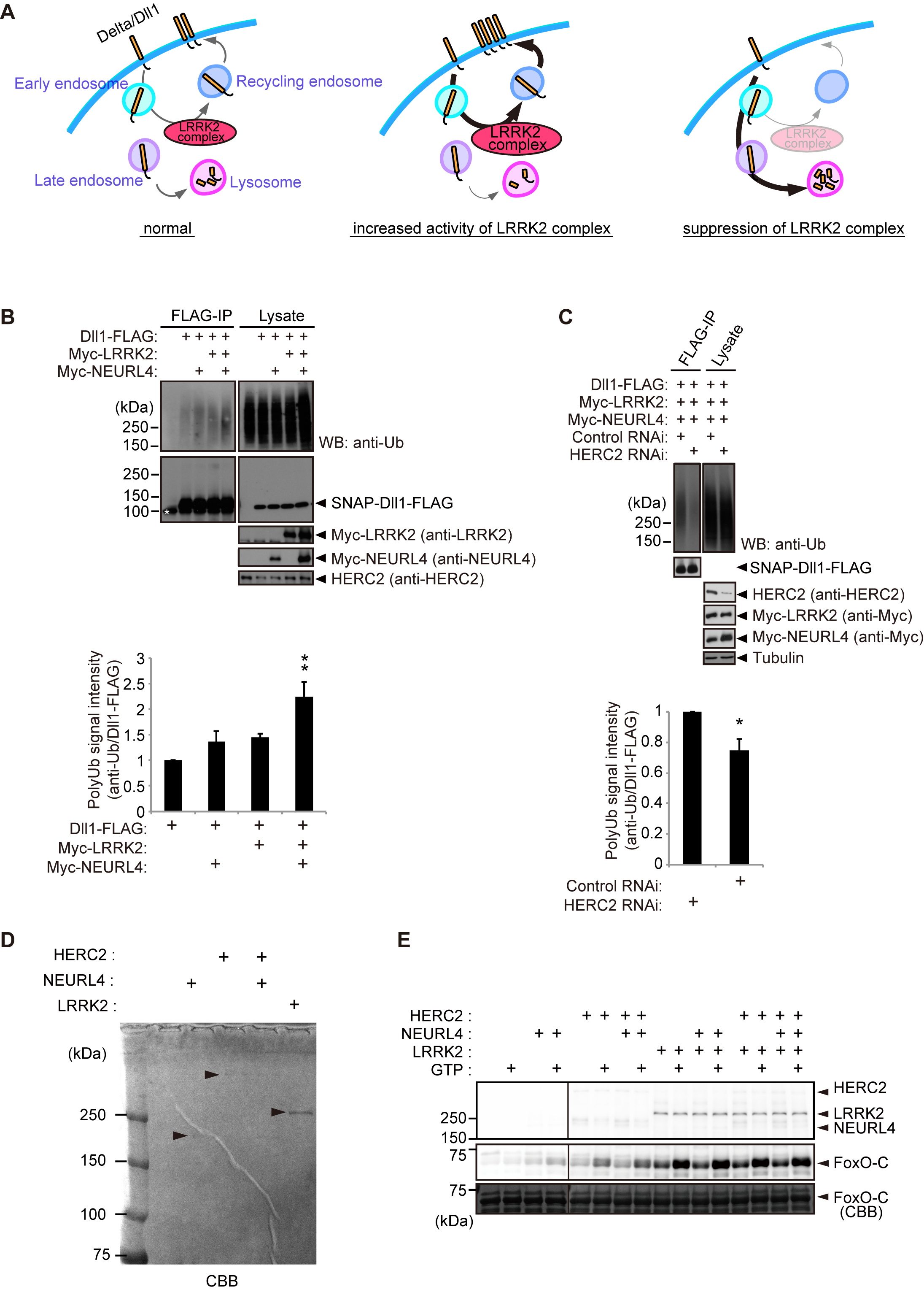

Supplement: S10 Fig — (A) LRRK2 complex stimulates endocytosis and recycling of Dl/Dll1. Increased activity of the LRRK2 complex or gain-of-function effects by the PD-associated mutation R1441G lead to the accumulation of Dl/Dll1, whereas loss-of-function of the LRRK2 complex results in Dl/Dll1 degradation via the late endosome-lysosome pathway. (B,C) The LRRK2 complex promotes Dll1 ubiquitination. HEK293T cell lysate transfected with the indicated cDNA and/or siRNA was subjected to immunoprecipitation with anti-FLAG antibody and analyzed by Western blotting with the indicated antibodies. The total amount of transfected cDNA was adjusted with vector DNA. ** p < 0.01 vs. Dll1-FLAG alone by one-way ANOVA, * p < 0.05 by Student’s t-test. Ub, ubiquitin. (D) FLAG-tagged LRRK2, HERC2 and NEURL4 were immunopurified using anti-FLAG beads and FLAG peptide. The amounts and purity were evaluated by CBB staining (arrowheads). (E) In vitro kinase assay of LRRK2. The indicated combination of proteins in the presence or absence of 2 mM GTP was added to kinase buffer containing recombinant GST-FoxO1-C as a LRRK2 substrate. Kinase assay by autoradiography was performed as described previously [55]. (TIF) [file pgen.1005503.s010.tif]
