# Supplementary material for: The Parkinson’s Disease-Associated Protein Kinase LRRK2 Modulates Notch Signaling through the Endosomal Pathway
Source: PLoS Genet. 2015 Sep 10;11(9):e1005503. doi: 10.1371/journal.pgen.1005503 (PMC4565672; doi:10.1371/journal.pgen.1005503)
Supplement: S1 Text — (DOCX) [file pgen.1005503.s011.docx]

**S1 Text. Antibodies used in this study**

The antibodies used for western blot analyses were as follows; rabbit anti-NEURL4 polyclonal antibody (raised against recombinant GST-NEURL4 [amino acids 970-1485] and also recognises Blue. 1:100); anti-LRRK2 (MJFF2, Abcam, 1:3,000); anti-GFP (GF090R, Nacalai Tesque, 1:1,000); anti-FLAG (M2, Sigma-Aldrich, 1:1,000); anti-Myc (#2272, Cell Signaling Technology, 1:1,000); anti-HA (3F10 or 12CA5, Roche Diagnostics, 1:1,000); anti-Notch1 (D6F11, Cell Signaling Technology, 1:1,000); anti-cleaved Notch1 (Val1744) (D3B8, Cell Signaling Technology, 1:1,000); anti-Dll1 (#ARP46625, AVIVA, 1:1,000); anti-HERC2 (A301-905A, Bethyl Laboratories, 1:2,000; #612366, BD, 1:500); anti-Tubulin (DM1A, Sigma-Aldrich, 1:1,000); anti-Actin (C4, Millipore, 1:10,000); anti-GAPDH (146C10, Cell Signaling Technology, 1:1,000); anti-ß-galactosidase (Z3781, Promega, 1:3,000). Rabbit anti-LRRK2 (1:1,000), anti-dLRRK (1:3,000) and anti-Ubiquitin (1:5,000) antibodies have been described previously [9,56,68]. Antibodies used for immunocyto/histochemical analyses were as follows: rabbit anti-NEURL4 antibody (Cosmo Bio, 1:2,000); anti-Hes1 polyclonal antibody (generated in Kageyama’s lab. 1:1,000); anti-FLAG (M2, Sigma-Aldrich, 1:500); anti-Myc (#2272, Cell Signaling Technology, 1:400), anti-Rab7 (Rab7-117, Sigma-Aldrich, 1:200), anti-HERC2 (A301-905A, Bethyl Laboratories, 1:500; #612366, BD, 1:100); anti-GFP (#632377, Clontech; A11122 and A10262, Life Technologies, 1:500); anti-Pax6 (Pax6, Developmental Studies Hybridoma Bank, 1:200); anti-TUJ1 (MMS-435-P, Covance, 1:500); anti-single stranded DNA (Dako Cytomation, 1:2,000); and anti-elav (9F8A9, Developmental Studies Hybridoma Bank, 1:100). Rabbit anti-*Drosophila* TH polyclonal antibody (1:250) has been described previously [9,56].
