# Supplementary material for: The Parkinson’s Disease-Associated Protein Kinase LRRK2 Modulates Notch Signaling through the Endosomal Pathway
Source: PLoS Genet. 2015 Sep 10;11(9):e1005503. doi: 10.1371/journal.pgen.1005503 (PMC4565672; doi:10.1371/journal.pgen.1005503)
Supplement: S2 Text — (DOCX) [file pgen.1005503.s012.docx]

**S2 Text. Fly genotypes used in this study**

**Fig. 3A**

*+/Y; Dpp-GAL4* (Dpp-GAL4)

*+/Y; UAS-Dl/+; Dpp-GAL4/+* (Dl)

*+/Y; UAS-Dl/; Dpp-GAL4, UAS-hLRRK2/+* (Dl, hLRRK2)

*+/Y; UAS-Dl/UAS-hLRRK2 RG; Dpp-GAL4* (Dl, hLRRK2^RG^)

*+/Y; UAS-Dl/; Dpp-GAL4, Blue^p{EPgy2}EY12221^/+* (Dl, Blue^EP^)

*dHERC2^p[45]G17171^/Y; UAS-Dl/+; Dpp-GAL4/+* (Dl, dHERC2^EP^)

**Fig. 3B**

*+/Y; UAS-Neur-∆RING/+; Dpp-GAL4/+* (Neur-∆RING)

*+/Y; UAS-Neur-∆RING/+; Dpp-GAL4, UAS-hLRRK2/+* (Neur-∆RING; hLRRK2)

*+/Y; UAS-Neur-∆ICD/UAS-hLRRK2 RG; Dpp-GAL4/+* (Neur-∆RING; hLRRK2^RG^)

*+/Y; UAS-Neur-∆ICD/UAS-hLRRK2 TN; Dpp-GAL4/+* (Neur-∆RING; hLRRK2^TN^)

*+/Y; UAS-Neur-∆RING/+; Dpp-GAL4, Blue^p{EPgy2}EY12221^/+* (Neur-∆RING; Blue^EP^)

*dHERC2^p[45]G17171^/Y; UAS-Neur-∆RING/+; Dpp-GAL4/+* (Neur-∆RING; dHERC2^EP^)

**Fig. 3C**

*Dl^3^/+* (Dl^+/-^)

*Dl^3^/Blue^47b-2^* (Dl^+/-^; Blue^+/47b-2^)

*Dl^3^/dLRRK^e03680^* (Dl^+/-^; dLRRK^+/-^)

**Fig. 3F-H**

*hs-Dl/+; UAS-Rab7-GFP/+; Dpp-GAL4/+* (Rab7; Control)

*hs-Dl/+; UAS-Rab7-GFP/UAS-dLRRK; Dpp-GAL4/+* (Rab7; dLRRK)

*hs-Dl/+; UAS-Rab7-GFP/+; Dpp-GAL4, Blue^p{EPgy2}EY12221^/+* (Rab7; Blue)

*hs-Dl/dHERC2^p[45]G17171^; UAS-Rab7-GFP/+; Dpp-GAL4/+* (Rab7; dHERC2)

*hs-Dl/+; UAS-Rab11-GFP/+; Dpp-GAL4/+* (Rab11; Control)

*hs-Dl/+; UAS-Rab11-GFP/UAS-dLRRK; Dpp-GAL4/+* (Rab11; dLRRK)

*hs-Dl/+; UAS-Rab11-GFP/+; Dpp-GAL4, Blue^p{EPgy2}EY12221^/+* (Rab11; Blue)

*hs-Dl/dHERC2^p[45]G17171^; UAS-Rab11-GFP/+; Dpp-GAL4/+* (Rab11; dHERC2)

**Fig. 3I**

*hs-Dl/+; UAS-Rab7-GFP/+; Dpp-GAL4/+* (Control)

*hs-Dl/+; UAS-Rab7-GFP/UAS-dLRRK; Dpp-GAL4/+* (dLRRK)

*hs-Dl/+; UAS-Rab7-GFP/+; Dpp-GAL4, Blue^p{EPgy2}EY12221^/+* (Blue)

*hs-Dl/dHERC2^p[45]G17171^; UAS-Rab7-GFP/+; Dpp-GAL4/+* (dHERC2)

**Fig. 7A,E Fig. 8A and S9A Fig**

*+/Y; ; UAS-white RNAi, tub-GAL80^ts^/TH-GAL4* (White^RNAi^),

*+/Y; ; UAS-Notch RNAi (VDRC1112)/+; tub-GAL80^ts^/TH-GAL4* (Notch RNAi #1),

*+/Y; ; UAS-Notch RNAi (VDRC27229), tub-GAL80^ts^/TH-GAL4* (Notch RNAi #2),

*+/Y; ; UAS-Dl RNAi (VDRC37288), tub-GAL80^ts^/TH-GAL4* (Dl RNAi #1),

*+/Y; ; UAS-Dl RNAi (VDRC37287), tub-GAL80^ts^/TH-GAL4* (Dl RNAi #2),

**Fig. 7B, Fig. 8A and S9A Fig**

*+/Y; ; UAS-mCD8::mRFP, tub-GAL80^ts^/TH-GAL4* (mRFP)

*+/Y; UAS-Notch/+; tub-GAL80^ts^/TH-GAL4* (Notch)

*+/Y; UAS-Dl/+; tub-GAL80^ts^/TH-GAL4* (Dl)

**Fig. 7C**

*elav-GAL4/Y; TH-GAL80; UAS-white RNAi, tub-GAL80^ts^* (White RNAi),

*elav-GAL4/Y; TH-GAL80/UAS-Notch RNAi* (#1, VDRC1112)*; tub-GAL80^ts^/+* (Notch RNAi #1),

*elav-GAL4/Y; TH-GAL80/+; UAS-Notch RNAi* (#2, VDRC27229)*, tub-GAL80^ts^/+*(Notch RNAi #2),

*elav-GAL4/Y; TH-GAL80/+; UAS-Dl RNAi* (#2, VDRC37288)*, tub-GAL80^ts^/+* (Dl RNAi #1),

*elav-GAL4/Y; TH-GAL80/+; UAS-Dl RNAi* (#2, VDRC37287)*, tub-GAL80^ts^/+* (Dl RNAi #2),

**Fig. 7D**

*elav-GAL4/Y; TH-GAL80/+; UAS-mCD8::mRFP, tub-GAL80^ts^/+* (mRFP)

*elav-GAL4/Y; UAS-Notch/TH-GAL80; tub-GAL80^ts^/+* (Notch)

*elav-GAL4/Y; UAS-Dl/TH-GAL80; tub-GAL80^ts^/+* (Dl)

*+/Y;; UAS-mCD8::mRFP/TH-GAL4* (mRFP)

*+/Y;; UAS-hLRRK2 WT/TH-GAL4* (hLRRK2 WT)

*+/Y;; UAS-hLRRK2 RG/+; TH-GAL4/+* (hLRRK2 RG)

*+/Y;; Blue^p{EPgy2}EY12221^/TH-GAL4* (Blue^EP^)

*dHERC2^p[45]G17171^/Y;; TH-GAL4/+* (dHERC2^EP^)

**Fig. 8B and S9B Fig**

*UAS-mCD8::mRFP/Y; UAS-Dl/+; tub-GAL80^ts^, TH-GAL4/+* (Dl, mRFP)

*+/Y; UAS-Dl/UAS- Neur; tub-GAL80^ts^, TH-GAL4/+* (Dl, Neur)

*+/Y; UAS-Dl/+; UAS-LacZ RNAi/tub-GAL80^ts^, TH-GAL4* (Dl, LacZ^RNAi^)

*+/Y; UAS-Dl/+; UAS-dLRRK RNAi/tub-GAL80^ts^, TH-GAL4* (Dl, dLRRK^RNAi^)

*+/Y; UAS-Dl/+; UAS-Blue RNAi/tub-GAL80^ts^, TH-GAL4* (Dl, Blue^RNAi^)

*+/Y; UAS-Dl/+; UAS-dHERC2 RNAi/tub-GAL80^ts^, TH-GAL4* (Dl, dHERC2^RNAi^)

**Fig. 8C and S9C Fig**

*UAS-mCD8::mRFP/Y;;TH-GAL4/+* (mRFP)

*+/Y;;TH-GAL4/UAS-hLRRK2* (LRRK2 WT)

*+/Y; UAS-hLRRK2 RG/+; TH-GAL4/+* (LRRK2 RG)

*+/Y;; TH-GAL4/Blue^p{EPgy2}EY12221^* (Blue^EP^)

*dHERC2^p[45]G17171^/Y;; TH-GAL4/ +* (dHERC2^EP^)

*UAS-mCD8::mRFP/Y;; UAS-LacZ RNAi/tub-GAL80^ts^, TH-GAL4* (mRFP, LacZ^RNAi^)

*UAS-mCD8::mRFP/Y;; UAS-Blue RNAi/tub-GAL80^ts^, TH-GAL4* (mRFP, Blue^RNAi^)

*UAS-mCD8::mRFP/Y;; UAS-dHERC2 RNAi/tub-GAL80^ts^, TH-GAL4* (mRFP, dHERC2 ^RNAi^)

*UAS-mCD8::mRFP/Y;; UAS-Dl RNAi/tub-GAL80^ts^, TH-GAL4* (mRFP, Dl^RNAi^)

*UAS-mCD8::mRFP/Y; UAS-hLRRK2 RG/+; UAS-LacZ RNAi/tub-GAL80^ts^, TH-GAL4* (LRRK2 RG, LacZ^RNAi^)

*UAS-mCD8::mRFP/Y; UAS-hLRRK2 RG/+; UAS-Blue RNAi/tub-GAL80^ts^, TH-GAL4* (LRRK2 RG, Blue^RNAi^)

*UAS-mCD8::mRFP/Y; UAS-hLRRK2 RG/+; UAS-dHERC2 RNAi/tub-GAL80^ts^, TH-GAL4* (LRRK2 RG, dHERC2^RNAi^)

*UAS-mCD8::mRFP/Y; UAS-hLRRK2 RG/+; UAS-Dl RNAi/tub-GAL80^ts^, TH-GAL4* (LRRK2 RG, Dl^RNAi^)

**S3A,B Fig**

*+/Y;; Da-GAL4/+* (Da-GAL4)

*+/Y;; Da-GAL4/Blue^p{EPgy2}EY12221^* (Da> Blue^EP^)

*+/Y;; Blue^p{EPgy2}EY12221^/+* (Blue^EP^/+)

*+/Y;; Blue^p{EPgy2}EY12221^/Blue^p{EPgy2}EY12221^* (Blue^EP^/Blue^EP^)

*+/Y* (Blue^+/+^)

*+/Y;; Blue^47b-2^/TM6B* (Blue^47b-2^/TM6B)

*+/Y;; Da-GAL4/+* (Da-GAL4)

*+/Y;; UAS-Blue RNAi; Da-GAL4/+* (Da>Blue RNAi)

**S3C,H Fig**

*+/Y;; Da-GAL4/+* (Da-GAL4)

*dHERC2^p[45]G17171^/Y;; Da-GAL4/+* (Da>dHERC2^EP^)

*+/Y;; Da-GAL4/UAS-dHERC2 RNAi* (Da>dHERC2^RNAi^)

**S3D Fig**

*+/Y; UAS-Neur-∆RING/+; Dpp-GAL4/+* (Neur-∆RING)

*+/Y; UAS-Neur-∆RING/+; Dpp-GAL4, dLRRK^e03680^/dLRRK^e03680^* (Neur-∆RING; dLRRK^-/-^)

*+/Y; UAS-Neur-∆RING/UAS-hLRRK2 GS; Dpp-GAL4/+* (Neur-∆RING; hLRRK2^GS^)

*+/Y; UAS-Neur-∆RING/UAS-hLRRK2 3KD; Dpp-GAL4/+* (Neur-∆RING; hLRRK2^KD^)

**S3E Fig**

*+/Y; UAS-GFP/+; Dpp-GAL4/UAS-GFP* (2x GFP)

*+/Y; UAS-Neur/+; Dpp-GAL4/UAS-GFP* (Neur; GFP)

*dHERC2^p[45]G17171^/Y; UAS-GFP/+; Dpp-GAL4/+* (GFP; dHERC2^EP^)

*dHERC2^p[45]G17171^/Y; UAS-Neur/+; Dpp-GAL4/+* (Neur; dHERC2^EP^)

**S3F Fig**

*+/Y;; Dpp-GAL4/UAS-Ser* (Ser)

*+/Y;; Dpp-GAL4, UAS-hLRRK2/UAS-Ser* (Ser; hLRRK2)

*+/Y;; Dpp-GAL4, Blue^p{EPgy2}EY12221^/UAS-Ser* (Ser; Blue^EP^)

*dHERC2^P[45]G17171^/Y;; Da-GAL4/UAS-Ser* (Ser; dHERC2^EP^)

**S3G Fig**

*N^55e11^/Y;; Dpp-GAL4/+* (N^55e11^)

*N^55e11^/Y;; Dpp-GAL4, UAS-hLRRK2/+* (N^55e11^; hLRRK2)

*N^55e11^/Y;; Dpp-GAL4, Blue^p{EPgy2}EY12221^/+* (N^55e11^; Blue^EP^)

*N^55e11^, dHERC2^p[45]G17171^;; Dpp-GAL4, Blue^p{EPgy2}EY12221^*/+ (N^55e11^; dHERC2^EP^)

*N^55e11^/Y;; Blue^47b-2^/+* (N^55e11^; Blue^47b-2^)

*N^55e11^/Y;; e03680/+* (N^55e11^; dLRRK^+/-^)

**S3I Fig**

*+/Y;; Da-GAL4/UAS-LacZ RNAi* (LacZ RNAi)

*+/Y;; Da-GAL4/UAS-dLRRK RNAi, VDRC22140* (dLRRK RNAi, 22140)

*+/Y;; Da-GAL4/UAS-dLRRK RNAi, VDRC 22139* (dLRRK RNAi, 22139)

*+/Y;; Da-GAL4/UAS-dLRRK* (dLRRK OE)

**S8 Fig**

*UAS-mCD8::mRFP/Y;UAS-TrpA1/+;tub-GAL80^ts^, TH-GAL4/N-LV, LexOP-dGFP* **(A, B)**, *+/Y;UAS-TrpA1/+;tub-GAL80^ts^,TH-GAL4/N-LV, LexOP-dGFP* (**C**, TrpA1), *+/Y;; UAS-shibire^ts1^,TH-GAL4/N-LV, LexOP-dGFP* (**C**, shi^ts1^)
